# Supplementary material for: Registered Report Protocol: Survey on attitudes and experiences regarding preregistration in psychological research
Source: PLoS One. 2021 Jul 2;16(7):e0253950. doi: 10.1371/journal.pone.0253950 (PMC8253437; doi:10.1371/journal.pone.0253950)
Supplement: S2 Text — The data collection procedure that is displayed in Fig 2 is further specified. Particularly, the specific procedures for collecting contact addresses as well as for inviting participants are described. (DOCX) [file pone.0253950.s002.docx]

Supporting information to ‘Registered Report Protocol: Survey on attitudes and experiences regarding preregistration in psychological research’:

**S2: Data collection procedure**

Lisa Spitzer^1^ & Stefanie Mueller^1^

^1^ Leibniz Institute for Psychology

## Specific procedure for collecting contact addresses

On each website (Web of Science, PubMed, PSYNDEX, PsycInfo, OSF Registries), specific rules will be followed for identifying potential participants’ email contacts:

On Web of Science, PubMed, PSYNDEX, and PsycInfo, the search term will be set to search “all fields”, it will be searched for the keyword “psychology”, and documents will be set to articles, to get a broad image of research articles focusing on psychology. Documents will be sorted from new to old. All articles that do not focus on psychology will be excluded.

On OSF Registries, it will also be searched for the keyword “psychology” and documents will be sorted from new to old. Only authors of regular preregistrations with focus on psychological research will be included. Preregistrations of Registered Reports will be excluded, as well as preregistrations of the “Election Research Preacceptance Competition” template as it has no psychological focus, and also other preregistrations that do not focus on psychology. Preregistrations that are based on the “OSF Standard Pre-Data Collection Preregistration” will also be excluded as this is oftentimes an almost empty preregistration. If preregistrations are double or empty, they will not be included. Additionally, documents that are clearly no preregistrations will also not be considered.

The identified information (source of work, title of work, year, broad topic of work, author name, email address, from which platform was the contact information taken from, link, and comments) will be coded in an a priori created coding sheet. Duplicate email addresses that may be sampled by different databases will be excluded.

## Specific procedure for inviting participants

During data collection, we will follow this specific procedure: In a first wave, *N* = 2960 persons whose email addresses will be identified as described above will be contacted with a personal email. Additionally, the survey will be advertised via social media and mailing lists throughout the whole time, pushing the ad every few days. All invited participants will be re-contacted after one week with a reminder email.

Two weeks after first invitation, quotas will be checked and if they are not filled yet, more members of open quotas will be recruited. For participants with a master’s degree, a doctoral degree, or a habilitation or full professorship, this will be done with the same procedure as described above, yet this time only contact addresses of members of the target quota will be collected. The here identified persons will be contacted and re-contacted after one week with a reminder. To determine how many people need to be recruited in the second wave to fill the quotas, we will use the response rate from the first invitation wave. This can be illustrated by an example: Since we are aiming for 25% quotas, our final sample should contain *n* = 74 participants from each quota (25% of our targeted sample size *N* = 296). If after the first invitation wave, only 64 members of a quota have participated, this would mean that ten people are still missing in this quota. To calculate how many more participants need to be invited to fill the quota, the overall response rate of the first invitation wave will be used: If only 237 (instead of 296) of the 2960 invited people have participated, this would correspond to an actual response rate of 8% instead of the estimated 10%. If we assume that 8% of invited researchers will also respond in the second invitation wave, we would need to invite 125 more participants to fill our example quota (since 10 is 8% of 125). Calculations of this kind will be done for all open quotas after the first invitation wave, to determine how many more participants to invite. However, due to reasons of practicality, no more than 740 more participants (i.e., 25% of the first invitation wave) will be invited per quota in the second wave. Meanwhile, if participants with a bachelor’s degree still need to be recruited this will be done via social media and mailing lists.

Two weeks after this second invitation wave, data collection will be stopped, thus approximately one month after initial invitation (i.e., one month plus the time it takes to gather more contact addresses for the second invitation wave). Thus, data collection will be stopped after participants of the first invitation wave have had the possibility to participate for one month (plus approximately one week), and participants of the second invitation wave have had two weeks to participate. If the optimal sample size is achieved before the end of this time frame, the survey will still be accessible to participants that were already invited until the end of the overall set time frame (one month plus approximately one week), but recruitment will be discontinued. Data collection will be stopped after this time frame even if the optimal *N* cannot be reached.
