# Supplementary material for: Registered Report Protocol: Survey on attitudes and experiences regarding preregistration in psychological research
Source: PLoS One. 2021 Jul 2;16(7):e0253950. doi: 10.1371/journal.pone.0253950 (PMC8253437; doi:10.1371/journal.pone.0253950)
Supplement: Attachment — Submitted filename: PONE-D-20-20445_reviewer_comments.pdf [file pone.0253950.s011.pdf]

PLOS ONE

Attitudes and experiences regarding preregistration in psychological research

--Manuscript Draft--

**Manuscript Number:**	PONE-D-20-20445
**Article Type:**	Registered Report Protocol
**Full Title:**	Attitudes and experiences regarding preregistration in psychological research
**Short Title:**	Attitudes and experiences regarding preregistration in psychology
**Corresponding Author:**	Lisa Spitzer, M. Sc.<br>Leibniz-Zentrum fur Psychologische Information und Dokumentation<br>Trier, GERMANY
**Keywords:**	preregistration; meta-research; psychology; open science; survey
**Abstract:**	Open science techniques aim to render research more transparent and reproducible and have gained increasing attention in recent years. One prominent open science technique is the preregistration of studies, which comprises creating and publicly sharing a research plan that is time-stamped and immutable before the data collection of a study has begun. Because of this technique's relevance for the improvement of research, its adoption in today's scientific community will be investigated. In particular, the focus will be on psychological science, as it has been a trailblazer to the open science development. Until now, no systematic evaluation of current attitudes and experiences regarding preregistration in psychology, as well as motivations and perceived obstacles has been conducted. To close this gap, the present study aims to investigate how psychological researchers currently think about preregistration, what they experienced when preregistering their studies, or what discourages them of doing so. Based on the theory of planned behavior, we reason that attitudes (moderated by the perceived importance of the topic of preregistration) as well as social norms and perceived behavioral control influence researchers' intention to preregister their studies. Furthermore, we plan to investigate differences between academic groups: As early career researchers are oftentimes seen as the driving force of the open science movement, while senior researchers are reported to be more reluctant, we expect differences in attitudes and experiences between these groups. By conducting this survey, we want to examine current attitudes and experiences of psychological researchers, in order to apply this knowledge to foster the practice of preregistration.
**Order of Authors:**	Lisa Spitzer, M. Sc.
	Stefanie Mueller
**Additional Information:**	
**Question**	**Response**
**Financial Disclosure**<br><br>Enter a financial disclosure statement that describes the sources of funding for the work included in this submission. Review the submission guidelines for detailed requirements. View published research articles from *PLOS ONE* for specific examples.<br><br>This statement is required for submission and **will appear in the published article** if the submission is accepted. Please make sure it is accurate.	The authors received no specific funding for this work.

Powered by Editorial Manager® and ProduXion Manager® from Aries Systems Corporation

**Unfunded studies**<br>Enter: *The author(s) received no specific funding for this work.*<br><br>**Funded studies**<br>Enter a statement with the following details:<br>• Initials of the authors who received each award<br>• Grant numbers awarded to each author<br>• The full name of each funder<br>• URL of each funder website<br>• Have or will the sponsors or funders play any role in the study design, data collection (when applicable) and analysis, decision to publish, or preparation of the manuscript?<br>• **NO** - Include this sentence at the end of your statement: *The funders had and will not have a role in study design, data collection and analysis, decision to publish, or preparation of the manuscript.*<br>• **YES** - Specify the role(s) played.<br><br>* typeset	
**Competing Interests**<br><br>Use the instructions below to enter a competing interest statement for this submission. On behalf of all authors, disclose any competing interests that could be perceived to bias this work—acknowledging all financial support and any other relevant financial or non-financial competing interests.<br><br>This statement **will appear in the published article** if the submission is accepted. Please make sure it is accurate. View published research articles from *PLOS ONE* for specific examples.	The authors have declared that no competing interests exist.

Powered by Editorial Manager® and ProduXion Manager® from Aries Systems Corporation

**NO authors have competing interests**<br><br>Enter: *The authors have declared that no competing interests exist.*<br><br>**Authors with competing interests**<br><br>Enter competing interest details beginning with this statement:<br><br>*I have read the journal's policy and the authors of this manuscript have the following competing interests: [insert competing interests here]*<br><br>* typeset	
**Ethics Statement**<br><br>Enter an ethics statement for this submission. This statement is required if the study involved:<br><br>• Human participants<br>• Human specimens or tissue<br>• Vertebrate animals or cephalopods<br>• Vertebrate embryos or tissues<br>• Field research<br><br>Write "N/A" if the submission does not require an ethics statement.<br><br>General guidance is provided below. Consult the submission guidelines for detailed instructions. **Make sure that all information entered here is included in the Methods section of the manuscript.**	The survey was approved by the ethics commission of Trier University, Germany (approval number: 07/2020). This form of consent was obtained in writing.

Powered by Editorial Manager® and ProduXion Manager® from Aries Systems Corporation

**Format for specific study types**<br><br>**Human Subject Research (involving human participants and/or tissue)**<br>• Give the name of the institutional review board or ethics committee that approved the study<br>• Include the approval number and/or a statement indicating approval of this research<br>• Indicate the form of consent obtained (written/oral) or the reason that consent was not obtained (e.g. the data were analyzed anonymously)<br><br>**Animal Research (involving vertebrate animals, embryos or tissues)**<br>• Provide the name of the Institutional Animal Care and Use Committee (IACUC) or other relevant ethics board that reviewed the study protocol, and indicate whether they approved this research or granted a formal waiver of ethical approval<br>• Include an approval number if one was obtained<br>• If the study involved *non-human primates*, add *additional details* about animal welfare and steps taken to ameliorate suffering<br>• If anesthesia, euthanasia, or any kind of animal sacrifice is part of the study, include briefly which substances and/or methods were applied<br><br>**Field Research**<br><br>Include the following details if this study involves the collection of plant, animal, or other materials from a natural setting:<br>• Field permit number<br>• Name of the institution or relevant body that granted permission	
**Data Availability**<br><br>Authors are required to make the data underlying their research findings fully available, without restriction.<br><br>If the manuscript reports pilot results, the data underlying those results should be made available at the time of publication. Submission of this Registered Report	Yes - any pilot data reported in this submission are fully available and data collected during the study will be made fully available without restriction upon study completion

Protocol implies a commitment that the data collected in the study, upon completion, will be made available with the Registered Report publication.<br><br>When legal or ethical requirements prohibit public data sharing, authors must indicate how researchers may obtain access to the data. See the PLOS Data Policy and FAQ for detailed information.<br><br>A Data Availability Statement describing where the data can be found is required at submission. Your answers to this question constitute the Data Availability Statement and **will be published in the article**, if accepted.<br><br>**Important:** Stating 'data available on request from the author' is not sufficient. If your data are only available upon request, select 'No' for the first question and explain your situation in the text box.<br><br>Do the authors confirm that all data underlying the findings of the study outlined in this Registered Report Protocol will be made fully available without restriction?	
**Describe where the data may be found in full sentences. If you are copying our sample text, replace any instances of XXX with the appropriate details.**<br><br>• If **no pilot data are reported in the manuscript**, please enter the following: *All relevant data from this study will be made available upon study completion.*<br>• If the manuscript reports pilot data **held in a public repository**, include URLs, accession numbers or DOIs. If this information will only be available after acceptance, indicate this by ticking the box below. For example: *All XXX files are available from the XXX database (accession number(s) XXX, XXX.).*	All relevant data from this study will be made available upon study completion.

Powered by Editorial Manager® and ProduXion Manager® from Aries Systems Corporation

• If the manuscript reports pilot data contained **within the manuscript and/or Supporting Information files**, enter the following:<br>*All relevant data are within the manuscript and its Supporting Information files.*<br>• If neither of these applies but you are able to provide **details of access elsewhere**, with or without limitations, please do so. For example:<br><br>*Data cannot be shared publicly because of [XXX]. Data are available from the XXX Institutional Data Access / Ethics Committee (contact via XXX) for researchers who meet the criteria for access to confidential data.*<br><br>*The data underlying the results presented in the study are available from (include the name of the third party and contact information or URL).*<br>• This text is appropriate if the data are owned by a third party and authors do not have permission to share the data.<br><br>* typeset	
Additional data availability information:	Tick here if the URLs/accession numbers/DOIs will be available only after acceptance of the manuscript for publication so that we can ensure their inclusion before publication.

Click here to access/download;Manuscript;manuscript_spitzer_mueller.docx

Attitudes and experiences regarding preregistration in psychological research

Lisa Spitzer[1*], Stefanie Mueller[1]

[1] Leibniz Institute for Psychology Information, Trier, Germany

* Corresponding author

E-mail: ls@leibniz-psychology.org (LS)

Abstract

Open science techniques aim to render research more transparent and reproducible and have gained increasing attention in recent years. One prominent open science technique is the preregistration of studies, which comprises creating and publicly sharing a research plan that is time-stamped and immutable before the data collection of a study has begun. Because of this technique's relevance for the improvement of research, its adoption in today's scientific community will be investigated. In particular, the focus will be on psychological science, as it has been a trailblazer to the open science development. Until now, no systematic evaluation of current attitudes and experiences regarding preregistration in psychology, as well as motivations and perceived obstacles has been conducted. To close this gap, the present study aims to investigate how psychological researchers currently think about preregistration, what they experienced when preregistering their studies, or what discourages them of doing so. Based on the theory of planned behavior, we reason that attitudes (moderated by the perceived importance of the topic of preregistration) as well as social norms and perceived behavioral control influence researchers' intention to preregister their studies. Furthermore, we plan to investigate differences between academic groups: As early career researchers are oftentimes seen as the driving force of the open science movement, while senior researchers are reported to be more reluctant, we expect differences in attitudes and experiences between these groups. By conducting this survey, we want to examine current attitudes and experiences of psychological researchers, in order to apply this knowledge to foster the practice of preregistration.

Introduction

Ever since Ioannidis [1] argued that most published research findings are false and a multitude of attempts failed to replicate previously significant effects (e.g., [2]), the reliability of published research findings has been a subject of discussion across all scientific disciplines. Summarizing these concerns, the term *reproducibility crisis* became known interdisciplinary [3]. When *Nature* [3] conducted a survey of more than 1500 researchers of multiple disciplines, 70% of researchers reported that they had failed to reproduce studies by others, and more than 50% had failed to reproduce their own studies. Across different sciences, 90% of researchers indicated their belief in a slight or even significant crisis [3].

Various reasons have been discussed for the high rate of false positive research results, and are oftentimes referred to as “questionable research practices” (e.g., see [4,5]). One example of such practices is the failure to control for biases, for example confirmation bias (more pronounced searching for and consideration of evidence that fits prior predictions in comparison to non-fitting evidence, e.g., see [6]) or hindsight bias (impression that the result was expected all along, e.g., see [7]). Low statistical power (e.g., due to small sample sizes) or poor quality control (e.g., no manipulation checks) are also problematic [6]. Other questionable research practices comprise selective reporting of dependent variables that yielded significant results or of experiments that confirmed the hypotheses, while not disclosing others [8,9], *p*-hacking (data dredging that makes it possible to present anything as significant [4]), or HARKing (hypothesizing after the results are known [7]). These are further associated with an insufficient distinction between prediction (confirmatory analyses) and post-diction (exploratory analyses) [9,10].

Yet, the existence and prevalence of questionable research practices should not be interpreted in the way that researchers use them voluntarily, but rather they are a product of us being human and prone to bias, a fact further amplified by the incentive structure in today's research culture [11]. In academia, researchers are presented with the alternatives to either "publish or perish" (e.g., see [12]). As the likelihood of a study being accepted by a journal is higher if the obtained results are novel and confirm the hypotheses – the so-called publication bias [6] – the incentive is high to (maybe unconsciously) alter the study so that it fits the required scheme. To counter these developments, the public, funding agencies as well as researchers themselves have called for *open science* techniques to make research more transparent and reproducible (e.g., see [6,13–15]).

Psychology's role in the open science movement

Limited reproducibility and questionable research practices have been identified as problematic in many sciences [3] and thus open science techniques have evolved in various disciplines. Yet, psychology seems to be an emphasized target of discussion (e.g., see [16]). On the one hand, the consequences of lacking reproducibility were experienced particularly drastic in psychology: Multiple large-scale research projects showed that published results could not be replicated [2,17], indications for a severe publication bias were identified [18], and questionable research practices were found to be highly prevalent in psychology [5]. On the other hand, due to this fact, psychological science has taken on a pioneering role in developing and implementing open science techniques [6,13,15]. An example for the influence of psychological open science initiatives on other research fields is the popularity of the Open Science Framework (OSF,

https://osf.io/). While it initially focused on psychological research, it is now an open science platform for all sciences. Because of psychology's impact on open science, the present survey focuses on psychologists' views on preregistration as one prominent open science technique.

Preregistration of studies to increase transparency

A preregistration is a research plan that is time-stamped, immutable, created before the data collection of a study has begun, and most often submitted to a public registry, thus making planned study details available to others [11,19,20]. The conciseness can range between a simple preregistration of the most basic elements (e.g., hypotheses) to rather detailed preregistrations containing questionnaires, experimental scripts and stimuli, or analysis code. A variety of templates are available (e.g., see https://aspredicted.org/; [20–22]). These are forms that list important elements to preregister and can be used by researchers to create their own preregistration. Reportedly, various templates might differ in their ability to decrease researcher degrees of freedom (i.e., flexibilities during data collection and analysis [23,24]).

Preregistration was first introduced to deal with two problems: publication bias and analytical flexibility (above all, selective reporting and p-hacking) [6]. Publication bias leads to the impression that there is stronger evidence for a phenomenon than actually exists since positive findings are represented disproportionately, whereas negative findings end up in a "file drawer" [6]. Analytical flexibility results from the fact that there is not only one way to analyze data in a given situation. Instead, there are many possible routes one might take to get a significant result (referred to as "garden of forking paths" [25]). When using null hypothesis significance testing (which is very prominent in various disciplines [11]), most often $\alpha = .05$ is used as significance

threshold to indicate that the given data is rather unlikely under the null hypothesis, and therefore there is sufficient evidence for the alternative hypothesis and thus for one's predictions. At the same time, this means that one accepts that a Type 1 error (a false positive result) might occur in 5% of analyses. It is problematic if the data influences statistical decisions (e.g., if multiple tests are conducted and only significant ones are regarded later, or if measures and statistical models applied to given data are determined based on already detected patterns in these data) as this leads to a higher probability for Type 1 errors for these tests [10,16,26]. Ultimately, this increases the rate of false positive findings in the research body and results in an over-estimation of effect sizes of phenomena [27].

Preregistration might be helpful against both of these problems: Publication bias might be decreased by preregistrations because they are publicly available and thus discoverable (see [20]). Preregistration also prioritizes theorizing and methods over results as the main criterion for judging the quality of research [20] which can further decrease publication bias (this might especially be the case for reviewed preregistrations, so-called Registered Reports [28]). Analytical flexibility is decreased by defining and documenting planned analyses beforehand because this way one can clearly distinguish confirmatory from exploratory analyses [9,11,20,29]. This might also decrease selective reporting [20], *p*-hacking and HARKing because all deviations from the a priori planned procedure are visible [29]. The preregistration can even serve as a plan to adhere to during study administration [11,20].

Adoption of preregistration in psychology

Preregistration is deemed important by a multitude of psychological researchers (e.g., see [6,9,11,29–32]). Nosek et al. [29] describe preregistration as “hard, and worthwhile”, while Wagenmakers and Dutilh [31] posit “seven selfish reasons for preregistration”. Overall, preregistering of studies is increasingly eminent in psychological research (as put in “Preregistration Becoming the Norm in Psychological Science” [30]).

Yet, it is still not completely common. In their recent study, Hardwicke et al. [33] found that only 3% of 188 examined articles from 2014 to 2017 included a preregistration statement. Stürmer et al. [34] also found that when early career researchers were asked about questionable research practices and open science, they deemed many open science practices necessary, yet towards preregistration compliance decreased: Only about half of the participants found that preregistration was fairly or very necessary, and even less indicated that they planned to consider preregistering their studies in the near future.

A number of reservations are mentioned frequently when discussing preregistration in psychology, including the fear that it leaves no flexibility during study administration and analysis and eliminates the possibility to conduct exploratory analyses (e.g., [11,20,35]). Some people are concerned that this might stifle discovery [36]. Others worry that someone might take their preregistered (and thus, publicly available) study idea and publish it before them (so-called scooping [35]). Additionally, the time costs and effort are often seen as obstacles regarding preregistration (e.g., see [20]). Besides these worries, some authors also express an overall critique regarding the concept of preregistration. Szollosi et al. [37–40] argue that preregistration is redundant when good theories are tested, and does not itself improve theories. Others argue that

preregistration cannot prevent some questionable research practices [41], or might not fit well with all types of research (see [20]). Moreover, some studies found problems with the current implementation of preregistration such as poor disclosure of deviations from preregistered plans in finished manuscripts [42]. Although most of the listed arguments against preregistration are counter-argued by supporters of preregistration (e.g., that exploratory analyses are still possible [11,20,35]) and evidence exists that preregistration significantly reduces questionable research practices and the rate of false positive findings (e.g., see [43,44] for examples of this effect in other fields), these reservations persist and some researchers remain skeptical.

Aim of this survey

While some surveys inquired about related topics, for example attitudes about open science or prevalence of questionable research practices in psychology [34,45,46], until now no systematic evaluation focusing on preregistration in psychology has been conducted. To close this gap, we propose the present survey investigating attitudes and experiences of psychological researchers towards preregistration. We want to examine the following questions: How do psychological researchers think of preregistration? Since no direct incentives are offered (as it is the case for Registered Reports that can obtain an in-principle-acceptance for publication by a journal [47]), do researchers view preregistration as an idealistic endeavor or do they report other, e.g., practical, motivations? What are perceived obstacles (and how can they be overcome)? To examine these questions, we will conduct an online survey among psychological students and researchers, focusing on overall attitudes as well as motivations and perceived obstacles regarding

preregistration. Obtaining a descriptive overview of the community's current attitudes and experiences will be the main focus of our survey.

Besides, we want to investigate two specific research questions: First, we want to examine which factors promote or prevent preregistration. According to the theory of planned behavior [48,49], the intention to perform a behavior can be seen as a direct antecedent of the actual behavior. In our specific context, this implies that the intention to preregister predicts whether researchers will actually preregister their studies or not. Because of this relevance, we want to investigate how such intentions are formed. As described by Ajzen and colleagues [48,49], three aspects influence intentions: 1) attitudes towards the behavior (which result from the ratio of perceived advantages to disadvantages of performing the behavior), 2) the subjective norm (which represents the perceived social pressure to perform or not perform the behavior), and 3) the perceived behavioral control (which focuses on the question if the subject has the resources and skills to perform the behavior or not; also see [50–55] for meta-analytical support of this model, and [56] for an overview). We will measure attitudes towards preregistration as well as subjective norms and perceived behavioral control, and investigate how they influence researchers' intention to preregister their studies in the future. Based on the model's postulations, we expect that more favorable attitudes and subjective norms as well as higher perceived behavioral control positively influence the intention to use preregistration. As the relative importance of attitudes, social norms and perceived behavioral control differs in dependence of considered behaviors, situations and populations [48,49], we want to test which of these is the strongest predictor for the intention to use preregistration. Additionally, we will also include perceived importance of preregistration as moderator for the strength of the influence of attitudes on intention. Such an extension of the model will compensate for potential non-attitudes (e.g., see [57]) and is explicitly allowed by the theory

of planned behavior [48,56]. Overall, we hypothesize that attitudes (moderated by the perceived importance of preregistration), social norms and perceived behavioral control can significantly predict researchers' intention to preregister their studies in the near future.

Second, we want to examine how attitudes, motivations and perceived obstacles differ between master students, PhD students, post-docs, and professors. We predict an overall difference between these academic groups. Moreover, we want to further inspect differences between early career researchers and more senior researchers. Early career researchers are oftentimes seen as the driving force of the open science movement (e.g., see [26]), and we want to investigate if their responses about preregistration indeed differ from those of other academic groups (as also found by Abele-Brehm et al. for hopes and fears regarding open science [45]).

Methods

Sampling procedure

Power analyses

As the focus will be on preregistration in psychology, data from psychological master students, PhD students, post-docs and professors will be collected (differentiation based on [45]). The sample size of N = 484 has been determined by using G*Power [58,59] in combination with a thorough review of the existing literature as described in the following paragraphs. The power analysis was specified to achieve a statistical power of 95% at a given significance threshold of 5%

and a type II error probability of also 5% ($\alpha = \beta = .05$). The here reported power analyses are available as supplementary material (see S1 Text).

To test which factors influence the intention to preregister (hypothesis 1), a multiple moderated regression model will be computed, based on the rationale of the theory of planned behavior [48,49,56]. Various meta-analyses have been conducted regarding this model, focusing on different topics (e.g., health behavior). In these, the percentage of variance of intention explained by attitudes, social norm and perceived behavioral control combined ranged between 30.4% < R^2 < 44.3% [50,52–55], from which the lowest reported effect size (R^2 = 30.4%) was chosen as minimal effect size of interest. The power analysis for the overall regression model (including all five predictors: attitudes, perceived importance of preregistration, attitudes x importance, social norm, perceived behavioral control) yielded an optimal sample size of $N = 52$ to achieve $\alpha = \beta = .05$. Additional power analyses were conducted to compute the optimal sample size to test the individual predictors. As comparable effect sizes, R^2 based on the averaged correlations of individual variables were searched for in the afore-mentioned meta-analyses, and the smallest ones were chosen for each power analysis. This resulted in an optimal sample size of $N = 42$ for testing attitudes, $N = 85$ for testing social norms, and $N = 236$ for testing perceived behavioral control as predictors for intention (with $\alpha = \beta = .05$).

Differences between academic groups (hypothesis 2) will be tested in a MANOVA. For this power analysis, the smallest reported effect size for group differences by Abele-Brehm et al. ($\eta^2 = .05$, [45] p. 4) was implemented as comparable effect size, and the lower bound of the 80% confidence interval was included as minimal effect size of interest (as recommended by [60,61]). This resulted in an optimal sample size of $N = 484$ to obtain $\alpha = \beta = .05$. As this is the highest computed necessary N, it will be used as overall optimal sample size.

Data collection and stopping rule

To ensure that all four groups (master students, PhD students, post-docs, and professors) are nearly equally represented in our final sample, a quota sampling will be used with a 25% quota for each subgroup. Yet, as it might be the case that some quotas might not be filled (e.g., that only few professors participate), quotas are defined for a slightly bigger sample than the necessary N = 484 to compensate for this eventuality. In particular, N = 700 will be the basis for our quotas which will therefore be n = 175 for each group. This enables us to reach the a priori computed power even if not all quotas can be filled, while still ensuring that no group is overrepresented (e.g., it won't be the case that 50% of the sample are students).

To recruit this sample, a method similar to the one by Field et al. [62] will be used. The term "psychology" will be searched for on OSF Registries (https://osf.io/registries) and Web of Science (https://apps.webofknowledge.com/) and resulting documents will be sorted from newest to oldest. These documents will be scanned for authors whose e-mail addresses will then be identified, not only including the first or corresponding author but all authors for whom contact information can be found via personal websites linked to OSF or Web of Science, or by searching for the author via Google, Google Scholar, Twitter, and ResearchGate. Detailed information about this procedure is given in the supplementary material (see S2 Text).

Response rates were rather low in many studies that focused on researchers as sample (e.g., 36.1% [5]; 36.1% [34]; 8.2% [45]; 45.9% [46]; 6% [62]; 17.6% [63]; 37.5% [64]). Based on these studies, the response rate of our survey is estimated to be around 26.77% (mean of the above). To compensate for a low response rate, a higher rate of invitations will be sent. In particular, N = 1810 persons will be invited (n = 905 via OSF Registries and n = 905 via Web of Science), as 1810 *

26.77% ≈ 484. This way even if the response rate is low (around 26.77%), the necessary N will still be reached.

During data collection, a specific procedure will be followed as described in Fig 1 (more specific information is given in the supplementary material, see S2 Text). Invitations will be sent to potential participants identified on OSF Registries and Web of Science in two waves. Individuals of the first wave will have approximately two months overall to participate in the survey, individuals of the second wave will have one month to participate. Both groups will receive a reminder e-mail two weeks after invitation. Additionally, the survey will be advertised on social media (Facebook, Twitter) and via student and researcher specific mailing lists, which will be seen as additional sources of participants. If the optimal sample size is achieved before the end of the set time frame (approximately two months), the survey will still be accessible to participants that were already invited, but recruitment will be discontinued.

Fig 1. Survey timeline and corresponding actions. The arrow illustrates the timeline while the boxes contain details about the sampling procedure.

As compensation, participants will have the possibility to participate in a lottery for 40 gift cards worth 50 € each. The survey was approved by the ethics commission of Trier University, Germany.

Exclusion and missing data

All participants that indicate that their research does not fall within the scope of psychology (or for master students, whose major is not psychology), or indicate that they are neither master student, PhD student, post-doc nor professor will be screened out at the beginning of the survey,

i.e., they will be directed to an exit page rather than to the main body of the survey. The same will apply for master students that indicate that they do not intend pursuing an academic career, as the survey targets a research oriented population.

At the end of the survey, participants will be asked whether they responded faithfully and participants who did not will also be excluded from the analyses. All remaining participants that completed all pages of the survey will be included in the final sample. Only these participants will be counted for fulfillment of quotas and planned sample size.

Material

The online survey was created with the software SoSci Survey (version 3.2.06) [65] and will be supplied via www.soscisurvey.de. Items of the survey can be categorized in eight categories: 1) sociodemographic questions, 2) items concerning the general usage of preregistration, 3) an *attitude* scale indicating the overall attitudes of participants regarding the concept of preregistration, 4) a *social norm* scale representing social norms and pressure, 5) a *perceived behavioral control* scale inquiring about researchers' perceived control over the potential preregistration of their studies, 6) items about motivations (a *motivation* scale plus additional more open items), 7) items about perceived obstacles (an *obstacle* scale plus additional more open items), and 8) suggestions for improving preregistration. Some items were adapted from other surveys that focused on related topics [34,45] and are complemented with additional items generated in reference to other theoretical and empirical works on preregistration and open science [41,62,64,66] and the theory of planned behavior [48,49,56]. The items of the present survey as

well as the original items including references are available as supplementary material (see S3 Table).

Different item formats will be included in the survey. Some items will be answered with answer scales, particularly four-point answer scales (1 = “Do not agree”, 2 = “Slightly disagree”, 3 = “Slightly agree”, 4 = “Strongly agree”, see [45]), or seven-point answer scales with labeled poles (1 = “Disagree”, 7 = “Agree”). Other items will include a single choice response format, still others a multiple choice response format. Whenever applicable, the options of these items are displayed in randomized order to eliminate potential sequence effects. Oftentimes, the given answer options to these items can be complemented with open text input (for most items, participants can select an “other” option and write their own answer). Yet other items request the participants to rank information based on perceived importance, or will be answered via open text input.

Procedure

Participants will receive the link to the survey via personal e-mail or social media call. After the welcoming page, participation information will be displayed and informed consent will be obtained. Then, participants will complete the main body of the survey which successively focuses on the different item categories (sociodemographic questions, general usage of preregistration, attitudes, social norms, perceived behavioral control, motivations, perceived obstacles, and suggestions for improvement). At the end of the survey, participants will have the option to participate in the lottery and to sign up for receiving a preprint of the survey results by entering their e-mail address, which will be saved separately from the other data. Lastly, a debriefing will

follow that explains the aim of the survey and the research questions that are investigated. Overall, completing the survey will take about 20 minutes. Screen recordings of the survey's procedure are available as supplementary material (see S4 and S5 Videos).

Data analysis

This Registered Report Protocol was written in *R Markdown* (version 2.2) [67,68]. R (version 4.0.1) [69] and the R-packages *afex* (version 0.27-2) [70], *Amelia* (version 1.7.6) [71], *beeswarm* (version 0.2.3) [72], *biotools* (version 3.1) [73], *car* (version 3.0-8) [74,75], *carData* (version 3.0-4) [75], *corrplot2017* (version 0.84) [76], *dplyr* (version 1.0.0) [77], *emmeans* (version 1.4.7) [78], *forcats* (version 0.5.0) [79], *ggplot2* (version 3.3.1) [80], *gplots* (version 3.0.3) [81], *heplots* (version 1.3-5) [82,83], *lattice* (version 0.20-41) [84], *lm.beta* (version 1.5-1) [85], *lme4* (version 1.1-23) [86], *MASS* (version 7.3-51.6) [87], *Matrix* (version 1.2-18) [88], *MBESS* (version 4.7.0) [89], *mlbench* (version 2.1-1) [90], *MVN* (version 5.8) [91], *olsrr* (version 0.5.3) [92], *papaja* (version 0.1.0.9942) [93], *popbio* (version 2.7) [94], *psych* (version 1.9.12.31) [95], *purrr* (version 0.3.4) [96], *Rcpp* (version 1.0.4.6) [97,98], *readr* (version 1.3.1) [99], *reshape2* (version 1.4.4) [100], *rpanel* (version 1.1-4) [101,102], *sp* (version 1.4-2) [103], *SpatialEpi* (version 1.2.3) [104], *stringr* (version 1.4.0) [105], *tibble* (version 3.0.1) [106], *tidyr* (version 1.1.0) [107], *tidyverse* (version 1.3.0) [108], *tkrplot* (version 0.0-24) [109], and *writexl* (version 1.3) [110] will be used for the analyses. Once the study has been conducted, StatTag (version 6.0.1) [111] will be used to insert the results calculated in R into the manuscript. Scripts for assumption testing and all analyses will be available as supplementary material (see S6 and S7 Scripts). Additionally, all data will be made publicly accessible after study completion.

Preprocessing

Data will be processed in the following way: Responses from multiple choice questions will be recoded (originally: 1 = “not checked” and 2 = “checked”; new: 0 = “not checked” and 1 = “checked”). Polarity of negatively poled items will be reversed. As a quality check, the attitude, social norm, perceived behavioral control, motivation and obstacle items will be inspected further and all items that show floor or ceiling effects will be excluded from the analyses (specifically, items for which ≥ 90% of participants indicated the lowest or highest category). Additionally, Cronbach’s alpha will be calculated as an indicator for reliability for each scale, and items for which Cronbach’s alpha of the scale is at least .10 higher if they are dropped will be excluded. The remaining items will be used to calculate mean scores for the attitude, social norm, perceived behavioral control, motivation, and obstacle scale respectively for each participant.

Descriptive analyses

After preprocessing the data, the overall response rate and quota fulfillment will be checked, participants’ overall comments will be assessed, and it will be investigated how long it took participants to complete the survey. Demographic information will be evaluated.

Then, the items that focus on general usage of, experiences with and suggestions for improving preregistration will be analyzed. For example, the overall proportion of participants that have preregistered before compared to those that have never preregistered before will be inspected, and it will be further analyzed if these proportions vary between academic groups. Additionally, participants’ ratings of perceived importance of preregistration as well as their intention to preregister their studies in the near future will be evaluated, and it will be inspected if this intention

varies between participants that have preregistered before versus have not preregistered before, and between academic groups.

Besides analyzing the items' given options, common themes mentioned in the open text inputs of our items will be identified and coded. First, words that were used very often will be analyzed quantitatively. Then in a more qualitative approach, coders will read the first 10% of responses and identify categories mentioned by the participants. These categories will then be transferred to new columns in a coding sheet and it will be coded if the category was mentioned in a response (= 1) or not (= 0). If new relevant topics appear to the coder that they have not coded before, these are added as categories as the coding continues, and coded later. This way, it will be possible to count the frequency of indication for common themes over all participants as well as compare it between different groups. Ambiguities will be discussed and solved in pairs. If no solution can be found, a third coder will be consulted. Results will be displayed as plots and tables to enable easy visual inspection of the frequencies for our report's readers.

Next, the attitude, social norm, perceived behavioral control, motivation, and obstacle scales will be inspected and the proportion of different answers for each item (*M*, *SD* and percent of answers on each scale degree) will be displayed in a table for easy inspection. Overall means for the attitude, social norm, perceived behavioral control, motivation, and obstacle scale as well as their standard deviations will be computed. Furthermore, the comments that participants could write to indicate a more elaborated opinion (as well as indicate other remarks) regarding these items will be considered.

Confirmatory analyses

A significance level of $\alpha = .05$ will be used for our confirmatory analyses. Before conducting statistical analyses, assumptions will be tested for each method. If assumptions are violated and tests are not robust against these violations, alternative methods will be used.

For hypothesis 1, we predict that attitudes, social norms and perceived behavioral control influence researchers' intention to preregister their studies in the near future. Perceived importance of the topic of preregistration will be included as moderator for attitudes. To test this, a multiple moderated regression model will be computed. Assumptions for this model include linearity, uncorrelated predictors, independence and normality of residuals, and homogeneity of variance. Linearity and uncorrelated predictors are assumed on a theoretical basis, as the fit of theory of planned behavior to predict intentions was tested successfully many times before [50–55]. Yet, there might be a higher multicollinearity for the product term (attitudes x perceived importance). To eliminate this potential problem, all predictor variables for our regression model will be centered. The independence of residuals is furthermore achieved by the survey's design. Normality of residuals and homogeneity of variance will be investigated by plotting the residuals (scatterplots, Q-Q plots and histograms). A violation of the normality of residuals is not impactful with high N, as it is the case with our sample, thus a violation would not be problematic. If the assumption of homogeneity of variance is violated, weighted least squares regression will be used instead of ordinary least squares. After testing these assumptions, the regression model will be computed: The answer to the item "I will preregister my studies in the near future." (seven-point scale: 1 = "Disagree", 7 = "Agree") will be included as dependent variable. As predictors, the attitude scale, perceived importance of preregistration, and their product term, social norm scale, and perceived behavioral control scale will be included. We expect that higher scores on the attitude scale (i.e.,

more positive attitudes), higher scores on the social norm scale (i.e., higher perceived social pressure), and higher scores on the perceived behavioral control scale (i.e., higher perceived control) are positive predictors for participants' intention to preregister their studies in the near future. Additionally, we expect that perceived importance of preregistration is itself a positive predictor for intention, and also significantly moderates the strength of the influence of attitudes. Furthermore, we expect that the overall model including all predictors can significantly predict researchers' intention to preregister their studies in the future. Because of these directional hypotheses, the regression weights will be tested in a one-tailed fashion. Standardized regression weights will be interpreted and compared.

The second hypothesis focuses on differences in attitudes, motivations and perceived obstacles between different academic groups. To investigate this prediction, a one-way MANOVA without repeated measures as well as three planned contrasts will be conducted. For the MANOVA, the attitude scale, motivation scale and obstacle scale will be included as dependent variables, while the academic group will serve as independent variable. For this model, the following assumptions will be tested: independence of observations, multivariate normality, and homogeneity of covariance matrices. The independence of observations might be violated due to our quota sampling, but since the quotas are defined to be bigger than the necessary *N* demands, participation in the survey is not as dependent on the participation rate of previous members of the quota as it would have been with stricter quotas. Multivariate normality will be tested for with the R-package *MVN* [91]. If this assumption is violated, the MANOVA can still be computed, but power might be lower than expected. This is compensated by our planned contrasts which will also investigate group differences (see below). Homogeneity of covariance matrices will be tested via a Box's M test. A violation is only impactful if groups are not evenly sized, which will be prevented by evenly

targeting our four subgroups and using a quota sampling. In case that violations occur that the MANOVA is not robust against (e.g., groups are not evenly sized and homogeneity of covariance matrices is violated), separate ANOVAs will be run to investigate if assumptions are fulfilled for some of the dependent variables. If this is not the case and assumptions are also violated for the separate ANOVAs, only the planned contrasts will be computed as they represent our most anticipated group differences (between PhD students and more senior researchers).

Specifically, we expect that there is a difference in attitudes as well as motivations and perceived obstacles between PhD students and more senior researchers. To investigate this, three planned contrasts will be conducted: scores on the attitude scale, motivation scale and obstacle scale will be compared between PhD students (weight = 1) versus post-docs (weight = –0.5) and professors (weight = –0.5). As our hypotheses are non-directional, the planned contrasts will be two-tailed tests. As three planned contrasts will be conducted to test one set of hypotheses, a Bonferroni-Holmes correction will be used to adjust the alpha level. For the planned contrasts, alpha will be $\alpha = .05 / (3 – 1) = .025$. As homogeneity of variances is an important assumption for planned contrasts, it will be tested with a Levene test. If variances are unequal, another approach for planned contrasts will be used that does not assume homogeneity of variance, as described by Maxwell and Delaney [112].

Acknowledgments

We are grateful to H. Bargon and A. Kuznik for their help with testing the survey, and their future help with recruitment and coding, and also to T. Dauber and J. Pauquet for testing the survey and proofreading.

References

1. Ioannidis JPA. Why most published research findings are false. PLoS medicine. 2005;2: e124. doi:10.1371/journal.pmed.0020124

2. Open Science Collaboration. Estimating the reproducibility of psychological science. Science (New York, NY). 2015;349: aac4716. doi:10.1126/science.aac4716

3. Baker M. 1,500 scientists lift the lid on reproducibility. Nature. 2016;533: 452–454. doi:10.1038/533452a

4. Simmons JP, Nelson LD, Simonsohn U. False-positive psychology: Undisclosed flexibility in data collection and analysis allows presenting anything as significant. Psychological science. 2011;22: 1359–1366. doi:10.1177/0956797611417632

5. John LK, Loewenstein G, Prelec D. Measuring the prevalence of questionable research practices with incentives for truth telling. Psychological science. 2012;23: 524–532. doi:10.1177/0956797611430953

6. Munafò MR, Nosek BA, Bishop DVM, Button KS, Chambers CD, Percie du Sert N, et al. A manifesto for reproducible science. Nature Human Behaviour. 2017;1: 0021. doi:10.1038/s41562-016-0021

7. Kerr NL. HARKing: Hypothesizing after the results are known. Personality and social psychology review : an official journal of the Society for Personality and Social Psychology, Inc. 1998;2: 196–217. doi:10.1207/s15327957pspr0203_4

8. Franco A, Malhotra N, Simonovits G. Underreporting in Psychology Experiments. Social Psychological and Personality Science. 2016;7: 8–12. doi:10.1177/1948550615598377

9. Wagenmakers E-J, Wetzels R, Borsboom D, van der Maas HLJ, Kievit RA. An Agenda for Purely Confirmatory Research. Perspectives on psychological science. 2012;7: 632–638. doi:10.1177/1745691612463078

10. Groot AD de. The meaning of “significance” for different types of research [translated and annotated by Eric-Jan Wagenmakers, Denny Borsboom, Josine Verhagen, Rogier Kievit, Marjan Bakker, Angelique Cramer, Dora Matzke, Don Mellenbergh, and Han L. J. van der Maas]. Acta Psychologica. 2014;148: 188–194. doi:10.1016/j.actpsy.2014.02.001

11. Nosek BA, Ebersole CR, DeHaven AC, Mellor DT. The preregistration revolution. Proceedings of the National Academy of Sciences of the United States of America. 2018;115: 2600–2606. doi:10.1073/pnas.1708274114

12. De Rond M, Miller AN. Publish or Perish: Bane or Boon of Academic Life? Journal of Management Inquiry. 2005;14: 321–329. doi:10.1177/1056492605276850

13. Nosek BA, Spies JR, Motyl M. Scientific Utopia: II. Restructuring Incentives and Practices to Promote Truth Over Publishability. Perspectives on psychological science. 2012;7: 615–631. doi:10.1177/1745691612459058

14. Nosek BA, Alter G, Banks GC, Borsboom D, Bowman SD, Breckler SJ, et al. Promoting an open research culture. Science (New York, NY). 2015;348: 1422–1425. doi:10.1126/science.aab2374

15. Spellman BA, Gilbert EA, Corker KS. Open Science. Stevens' Handbook of Experimental Psychology and Cognitive Neuroscience. Hoboken, New Jersey: John Wiley & Sons; 2018. pp. 1–47. doi:10.1002/9781119170174.epcn519

16. Shrout PE, Rodgers JL. Psychology, Science, and Knowledge Construction: Broadening Perspectives from the Replication Crisis. Annual review of psychology. 2018;69: 487–510. doi:10.1146/annurev-psych-122216-011845

17. Baker M. Over half of psychology studies fail reproducibility test. 2015. doi:10.1038/nature.2015.18248

18. Fanelli D. "Positive" results increase down the Hierarchy of the Sciences. PLoS ONE. 2010;5: e10068. doi:10.1371/journal.pone.0010068

19. DeHaven AC, Bowman S. The What, Why, and How of Preregistration [Video]. 2020. Available from: https://www.youtube.com/watch?v=8QK2-udwoK8

20. van 't Veer AE, Giner-Sorolla R. Pre-registration in social psychology—A discussion and suggested template. Journal of Experimental Social Psychology. 2016;67: 2–12. doi:10.1016/j.jesp.2016.03.004

21. Brandt MJ, IJzerman H, Dijksterhuis A, Farach FJ, Geller J, Giner-Sorolla R, et al. The Replication Recipe: What makes for a convincing replication? Journal of Experimental Social Psychology. 2014;50: 217–224. doi:10.1016/j.jesp.2013.10.005

22. van den Akker O, Weston SJ, Campbell L, Chopik WJ, Damian RI, Davis-Kean P, et al. Preregistration of secondary data analysis: A template and tutorial. PsyArXiv [Preprint]. 2019 [posted 2019 Nov 20; cited 2020 July 02]. doi:10.31234/osf.io/hvfmr

23. Wicherts JM, Veldkamp CLS, Augusteijn HEM, Bakker M, van Aert RCM, van Assen MALM. Degrees of Freedom in Planning, Running, Analyzing, and Reporting Psychological Studies: A Checklist to Avoid *p*-Hacking. Frontiers in psychology. 2016;7: 1832. doi:10.3389/fpsyg.2016.01832

24. Veldkamp CLS, Bakker M, van Assen MALM, Crompvoets EAV, Ong HH, Nosek BA, et al. Ensuring the quality and specificity of preregistrations PsyArXiv [Preprint]. 2018 [posted 2018 Sep 04; revised 2018 Sep 05; revised 2018 Sep 19; revised 2020 Feb 27; cited 2020 July 02]. doi:10.31234/osf.io/cdgyh

25. Gelman A, Loken E. The Statistical Crisis in Science: Data-dependent analysis— a "garden of forking paths" — explains why many statistically significant comparisons don't hold up. American Scientist. 2014;102: 460–465. doi:10.1511/2014.111.460

26. Chambers C. The seven deadly sins of psychology: A manifesto for reforming the culture of scientific practice. First paperback edition. Princeton, New Jersey; Woodstock, Oxfordshire: Princeton University Press; 2019.

27. Lindsay DS. Arguments for Preregistering Psychology Research. Psychonomic Society. 2019. Available from: https://featuredcontent.psychonomic.org/arguments-for-preregistering-psychology-research/

28. Chambers CD. Registered reports: A new publishing initiative at Cortex. Cortex. 2013;49: 609–610. doi:10.1016/j.cortex.2012.12.016

29. Nosek BA, Beck ED, Campbell L, Flake JK, Hardwicke TE, Mellor DT, et al. Preregistration Is Hard, And Worthwhile. Trends in cognitive sciences. 2019;23: 815–818. doi:10.1016/j.tics.2019.07.009

30. Nosek BA, Lindsay DS. Preregistration Becoming the Norm in Psychological Science. Association for Psychological Science. 2018. Available from: https://www.psychologicalscience.org/observer/preregistration-becoming-the-norm-in-psychological-science/comment-page-1

31. Wagenmakers E-J, Dutilh G. Seven Selfish Reasons for Preregistration. Association for Psychological Science. 2016;29. Available from: https://www.psychologicalscience.org/observer/seven%C3%A2%E2%82%AC%20selfish%C3%A2%E2%82%AC%20reasons%C3%A2%E2%82%AC%20for%C3%A2%E2%82%AC%20preregistration

32. Klein O, Hardwicke TE, Aust F, Breuer J, Danielsson H, Hofelich Mohr A, et al. A Practical Guide for Transparency in Psychological Science. Collabra: Psychology. 2018;4: 20. doi:10.1525/collabra.158

33. Hardwicke TE, Thibault RT, Kosie JE, Wallach JD, Kidwell M, Ioannidis J. Estimating the prevalence of transparency and reproducibility-related research practices in psychology (2014-2017). MetaArXiv [Preprint]. 2020 [posted 2020 Jan 02; cited 2020 July 02]. doi:10.31222/osf.io/9sz2y

34. Stürmer S, Oeberst A, Trötschel R, Decker O. Early-Career Researchers' Perceptions of the Prevalence of Questionable Research Practices, Potential Causes, and Open Science. Social Psychology. 2017;48: 365–371. doi:10.1027/1864-9335/a000324

35. Nosek BA. Preregistration: Improve Research Rigor, Reduce Bias [Video]. 2018. Available from: https://www.youtube.com/watch?v=PboPpcg6ik4

36. Goldin-Meadow S. Why Preregistration Makes Me Nervous. Association for Psychological Science. 2016. Available from: psychologicalscience.org/observer/why-preregistration-makes-me-nervous/comment-page-1

37. Szollosi A, Kellen D, Navarro D, Shiffrin R, Rooij I, Zandt T, et al. Preregistration is redundant, at best. PsyArXiv [Preprint]. 2019 [posted 2019 Oct 31; revised 2019 Nov 26; cited 2020 July 02]. doi:10.31234/osf.io/x36pz

38. Szollosi A, Donkin C. Arrested theory development: The misguided distinction between exploratory and confirmatory research. PsyArXiv [Preprint]. 2019 [posted 2019 Sep 21; cited 2020 July 02]. doi:10.31234/osf.io/suzej

39. Szollosi A, Donkin C. Neglected Sources of Flexibility in Psychological Theories: From Replicability to Good Explanations. Computational Brain & Behavior. 2019;2: 190–192. doi:10.1007/s42113-019-00045-y

40. Szollosi A, Kellen D, Navarro DJ, Shiffrin R, van Rooij I, van Zandt T, et al. Is Preregistration Worthwhile? Trends in cognitive sciences. 2020;24: 94–95. doi:10.1016/j.tics.2019.11.009

41. Ikeda A, Xu H, Fuji N, Zhu S, Yamada Y. Questionable research practices following pre-registration. Japanese Psychological Review. 2019;62: 281–295.

42. Claesen A, Gomes SLBT, Tuerlinckx F, Vanpaemel W. Preregistration: Comparing Dream to Reality. PsyArXiv [Preprint]. 2019 [posted 2019 May 09; revised 2019 May 12; cited 2020 July 02]. doi:10.31234/osf.io/d8wex

43. Kaplan RM, Irvin VL. Likelihood of Null Effects of Large NHLBI Clinical Trials Has Increased over Time. PLoS ONE. 2015;10: e0132382. doi:10.1371/journal.pone.0132382

44. Swaen GG, Teggeler O, van Amelsvoort LG. False positive outcomes and design characteristics in occupational cancer epidemiology studies. International journal of epidemiology. 2001;30: 948–954. doi:10.1093/ije/30.5.948

45. Abele-Brehm AE, Gollwitzer M, Steinberg U, Schönbrodt FD. Attitudes Toward Open Science and Public Data Sharing. Social Psychology. 2019;50: 252–260. doi:10.1027/1864-9335/a000384

46. DeHaven AC, Graf C, Mellor DT, Morris E, Moylan E, Pedder S, et al. Registered Reports: Views from editors, reviewers and authors. PsyArXiv [Preprint]. 2019 [posted 2019 Sep 17; revised 2019 Sep 17; cited 2020 July 02. doi:10.31222/osf.io/ndvek

47. Chambers CD, Dienes Z, McIntosh RD, Rotshtein P, Willmes K. Registered reports: Realigning incentives in scientific publishing. Cortex. 2015;66: A1–2. doi:10.1016/j.cortex.2015.03.022

48. Ajzen I. The theory of planned behavior. Organizational behavior and human decision processes. 1991;50: 179–211. doi:10.1016/0749-5978(91)90020-T

49. Ajzen I, Fishbein M, Lohmann, Albarracín D. The influence of attitudes on behavior. In: Albarracín D, Johnson BT, editors. The handbook of attitudes: Volume 1: Basic principles (2nd Ed). New York: Routledge; 2019. pp. 197–255.

50. Armitage CJ, Conner M. Efficacy of the Theory of Planned Behaviour: A meta-analytic review. The British journal of social psychology. 2001;40: 471–499. doi:10.1348/014466601164939

51. Notani A. Moderators of Perceived Behavioral Control's Predictiveness in the Theory of Planned Behavior: A Meta-Analysis. Journal of Consumer Psychology. 1998;7: 247–271. doi:10.1207/s15327663jcp0703_02

52. Rivis A, Sheeran P. Descriptive norms as an additional predictor in the theory of planned behaviour: A meta-analysis. Current Psychology. 2003;22: 218–233. doi:10.1007/s12144-003-1018-2

53. McEachan RRC, Conner M, Taylor NJ, Lawton RJ. Prospective prediction of health-related behaviours with the Theory of Planned Behaviour: A meta-analysis. Health Psychology Review. 2011;5: 97–144. doi:10.1080/17437199.2010.521684

54. Godin G, Kok G. The theory of planned behavior: A review of its applications to health-related behaviors. American journal of health promotion. 1996;11: 87–98. doi:10.4278/0890-1171-11.2.87

55. Downs DS, Hausenblas HA. The Theories of Reasoned Action and Planned Behavior Applied to Exercise: A Meta-analytic Update. Journal of Physical Activity and Health. 2005;2: 76–97. doi:10.1123/jpah.2.1.76

56. Ajzen I. The theory of planned behaviour: Reactions and reflections. Psychology & health. 2011;26: 1113–1127. doi:10.1080/08870446.2011.613995

57. Schuman H, Presser S. Questions and answers in attitude surveys: Experiments on question form, wording, and context. New York: Academic Press; 1981.

58. Faul F, Erdfelder E, Lang A-G, Buchner A. G*Power 3: A flexible statistical power analysis program for the social, behavioral, and biomedical sciences. Behavior research methods. 2007;39: 175–191. doi:10.3758/BF03193146

59. Faul F, Erdfelder E, Buchner A, Lang A-G. Statistical power analyses using G*Power 3.1: Tests for correlation and regression analyses. Behavior research methods. 2009;41: 1149–1160. doi:10.3758/BRM.41.4.1149

60. Perugini M, Gallucci M, Costantini G. Safeguard Power as a Protection Against Imprecise Power Estimates. Perspectives on psychological science. 2014;9: 319–332. doi:10.1177/1745691614528519

61. Albers C, Lakens D. When power analyses based on pilot data are biased: Inaccurate effect size estimators and follow-up bias. Journal of Experimental Social Psychology. 2018;74: 187–195. doi:10.1016/j.jesp.2017.09.004

62. Field SM, Wagenmakers E-J, Kiers HAL, Hoekstra R, Ernst AF, van Ravenzwaaij D. The effect of preregistration on trust in empirical research findings: Results of a registered report. Royal Society open science. 2020;7: 181351. doi:10.1098/rsos.181351

63. van den Akker O, Alvarez LD, Bakker M, Wicherts JM, van Assen MALM. How do academics assess the results of multiple experiments? PsyArXiv [Preprint]. 2018 [posted 2018 Dec 16; cited 2020 July 02]. doi:10.31234/osf.io/xyks4

64. Mathieu S, Chan A-W, Ravaud P. Use of trial register information during the peer review process. PLoS ONE. 2013;8: e59910. doi:10.1371/journal.pone.0059910

65. Leiner DJ. SoSci Survey (Version 3.2.06) [Computer software]. 2019. Available from: https://www.soscisurvey.de

66. Allen C, Mehler DMA. Open science challenges, benefits and tips in early career and beyond. PLoS biology. 2019;17: e3000246. doi:10.1371/journal.pbio.3000246

67. Xie Y, Allaire JJ, Grolemund G. R Markdown: The Definitive Guide. Boca Raton, Florida: Chapman and Hall/CRC; 2018. Available from: https://bookdown.org/yihui/rmarkdown

68. Allaire JJ, Xie Y, McPherson J, Luraschi J, Ushey K, Atkins A, et al. rmarkdown: Dynamic Documents for R. Version 2.2 [software]. 2020 [cited 2020 July 02]. Available from: https://github.com/rstudio/rmarkdown

69. R Core Team. R: A language and environment for statistical computing. Vienna, Austria: R Foundation for Statistical Computing; 2020. Available from: https://www.R-project.org/

70. Singmann H, Bolker B, Westfall J, Aust F, Ben-Shachar MS. Afex: Analysis of factorial experiments. Version 0.27-2 [software]. 2020 [cited 2020 July 02]. Available from: https://CRAN.R-project.org/package=afex

71. Honaker J, King G, Blackwell M. Amelia II: A program for missing data. Journal of Statistical Software. 2011;45: 1–47. Available from: http://www.jstatsoft.org/v45/i07/

72. Eklund A. Beeswarm: The bee swarm plot, an alternative to stripchart. Version 0.2.3 [software]. 2016 [cited 2020 July 02]. Available from: https://CRAN.R-project.org/package=beeswarm

73. Silva AR da, Malafaia G, de Menezes IPP. Biotools: An r function to predict spatial gene diversity via an individual-based approach. Genetics and Molecular Research. 2017;16: gmr16029655.

74. Fox J, Weisberg S. An R companion to applied regression. Third. Thousand Oaks CA: Sage; 2019. Available from: https://socialsciences.mcmaster.ca/jfox/Books/Companion/

75. Fox J, Weisberg S, Price B. CarData: Companion to applied regression data sets. Version 3.0-4 [software]. 2019 [cited 2020 July 02]. Available from: https://CRAN.R-project.org/package=carData

76. Wei T, Simko V. R package "corrplot": Visualization of a correlation matrix. Version 0.84 [software]. 2017 [cited 2020 July 02]. Available from: https://github.com/taiyun/corrplot

77. Wickham H, François R, Henry L, Müller K. Dplyr: A grammar of data manipulation. Version 1.0.0 [software]. 2020 [cited 2020 July 02]. Available from: https://CRAN.R-project.org/package=dplyr

78. Lenth R. Emmeans: Estimated marginal means, aka least-squares means. Version 1.4.7 [software]. 2020 [cited 2020 July 02]. Available from: https://CRAN.R-project.org/package=emmeans

79. Wickham H. Forcats: Tools for working with categorical variables (factors). Version 0.5.0 [software]. 2020 [cited 2020 July 02]. Available from: https://CRAN.R-project.org/package=forcats

80. Wickham H. Ggplot2: Elegant graphics for data analysis. Springer-Verlag New York; 2016. Available from: https://ggplot2.tidyverse.org

81. Warnes GR, Bolker B, Bonebakker L, Gentleman R, Huber W, Liaw A, et al. Gplots: Various r programming tools for plotting data. Version 3.0.3 [software]. 2020 [cited 2020 July 02]. Available from: https://CRAN.R-project.org/package=gplots

82. Friendly M. HE plots for multivariate general linear models. Journal of Computational and Graphical Statistics. 2007;16: 421–444.

83. Friendly M. HE plots for repeated measures designs. Journal of Statistical Software. 2010;37: 1–40. Available from: http://www.jstatsoft.org/v37/i04/

84. Sarkar D. Lattice: Multivariate data visualization with r. New York: Springer; 2008. Available from: http://lmdvr.r-forge.r-project.org

85. Behrendt S. Lm.beta: Add standardized regression coefficients to lm-objects. Version 1.5-1 [software]. 2014 [cited 2020 July 02]. Available from: https://CRAN.R-project.org/package=lm.beta

86. Bates D, Mächler M, Bolker B, Walker S. Fitting linear mixed-effects models using lme4. Journal of Statistical Software. 2015;67: 1–48. doi:10.18637/jss.v067.i01

87. Venables WN, Ripley BD. Modern applied statistics with s. Fourth. New York: Springer; 2002. Available from: http://www.stats.ox.ac.uk/pub/MASS4

88. Bates D, Maechler M. Matrix: Sparse and dense matrix classes and methods. Version 1.2-18 [software]. 2019 [cited 2020 July 02]. Available from: https://CRAN.R-project.org/package=Matrix

89. Kelley K. MBESS: The mbess r package. Version 4.7.0 [software]. 2020 [cited 2020 July 02]. Available from: https://CRAN.R-project.org/package=MBESS

90. Leisch F, Dimitriadou E. Mlbench: Machine learning benchmark problems. Version 2.1-1 [software]. 2010 [cited 2020 July 02]. Available from: https://CRAN.R-project.org/web/packages/mlbench/index.html

91. Korkmaz S, Goksuluk D, Zararsiz G. MVN: An r package for assessing multivariate normality. The R Journal. 2014;6: 151–162. Available from: https://journal.r-project.org/archive/2014-2/korkmaz-goksuluk-zararsiz.pdf

92. Hebbali A. Olsrr: Tools for building ols regression models. Version 0.5.3 [software]. 2020 [cited 2020 July 02]. Available from: https://CRAN.R-project.org/package=olsrr

93. Aust F, Barth M. papaja: Create APA manuscripts with R Markdown. Version 0.1.0.9942 [software]. 2020 [cited 2020 July 02]. Available from: https://github.com/crsh/papaja

94. Stubben CJ, Milligan BG. Estimating and analyzing demographic models using the popbio package in r. Journal of Statistical Software. 2007;22. doi:10.18637/jss.v022.i11

95. Revelle W. Psych: Procedures for psychological, psychometric, and personality research. Version 1.9.12.31 [software]. Evanston, Illinois: Northwestern University; 2019 [cited 2020 July 02]. Available from: https://CRAN.R-project.org/package=psych

96. Henry L, Wickham H. Purrr: Functional programming tools. Version 0.3.4 [software]. 2019 [cited 2020 July 02]. Available from: https://CRAN.R-project.org/package=purrr

97. Eddelbuettel D, François R. Rcpp: Seamless R and C++ integration. Journal of Statistical Software. 2011;40: 1–18. doi:10.18637/jss.v040.i08

98. Eddelbuettel D, Balamuta JJ. Extending extitR with extitC++: A Brief Introduction to extitRcpp. PeerJ Preprints. 2017;5: e3188v1. doi:10.7287/peerj.preprints.3188v1

99. Wickham H, Hester J, Francois R. Readr: Read rectangular text data. Version 1.3.1 [software]. 2018 [cited 2020 July 02]. Available from: https://CRAN.R-project.org/package=readr

100. Wickham H. Reshaping data with the reshape package. Journal of Statistical Software. 2007;21: 1–20. Available from: http://www.jstatsoft.org/v21/i12/

101. Bowman A, Crawford E, Alexander G, Bowman RW. rpanel: Simple interactive controls for R functions using the tcltk package. Journal of Statistical Software. 2007;17: 1–18. Available from: http://www.jstatsoft.org/v17/i09/

102. Bowman AW, Gibson I, Scott EM, Crawford E. Interactive teaching tools for spatial sampling. Journal of Statistical Software. 2010;36: 1–17. Available from: http://www.jstatsoft.org/v36/i13/

103. Pebesma EJ, Bivand RS. Classes and methods for spatial data in R. R News. 2005;5: 9–13. Available from: https://CRAN.R-project.org/doc/Rnews/

104. Kim AY, Wakefield J. SpatialEpi: Methods and data for spatial epidemiology. Version 1.2.3 [software]. 2018 [cited 2020 July 02]. Available from: https://CRAN.R-project.org/package=SpatialEpi

105. Wickham H. Stringr: Simple, consistent wrappers for common string operations. Version 1.4.0 [software]. 2019 [cited 2020 July 02]. Available from: https://CRAN.R-project.org/package=stringr

106. Müller K, Wickham H. Tibble: Simple data frames. Version 3.0.1 [software]. 2020 [cited 2020 July 02]. Available from: https://CRAN.R-project.org/package=tibble

107. Wickham H, Henry L. Tidyr: Tidy messy data. Version 1.1.0 [software]. 2020 [cited 2020 July 02]. Available from: https://CRAN.R-project.org/package=tidyr

108. Wickham H, Averick M, Bryan J, Chang W, McGowan LD, François R, et al. Welcome to the tidyverse. Journal of Open Source Software. 2019;4: 1686. doi:10.21105/joss.01686

109. Tierney L. Tkrplot: TK rplot. Version 0.0-24 [software]. 2018 [cited 2020 July 02]. Available from: https://CRAN.R-project.org/package=tkrplot

110. Ooms J. Writexl: Export data frames to excel ’xlsx’ format. Version 1.3 [software]. 2019 [cited 2020 July 02]. Available from: https://CRAN.R-project.org/package=writexl

111. Welty LJ, Rasmussen LV, Baldridge AS, Whitley E. StatTag. Version 6.0.1 [software]. 2020 [cited 2020 July 02]. Chicago, Illinois, United States: Galter Health Sciences Library. 2016. doi:10.18131/G36K76

112. Maxwell SE, Delaney HD, Kelley K. Designing experiments and analyzing data: A model comparison perspective. Third edition. New York, NY: Routledge, Taylor & Francis Group; 2018.

Supporting information

S1 Text. Power analyses. Power analyses for both hypotheses are presented here. For hypothesis 1, power analyses were performed for the entire model as well as for the individual predictors.

S2 Text. Data collection procedure. The data collection procedure that is displayed in Fig 1 is further specified. Particularly, the specific procedures for collecting contact addresses as well as for inviting participants are described.

S3 Table. Survey items. The items of the survey are presented. If they were derived from other studies, the original items as well as references are given.

S4 Video. Screen recording of the survey procedure (version A). This screen recording shows the procedure of the survey for participants that have answered the item “Have you preregistered a study before?” with “yes” (as this is a filter question for some of the following items).

S5 Video. Screen recording of the survey procedure (version B). This screen recording shows the procedure of the survey for participants that have answered the item “Have you preregistered a study before?” with “no” (as this is a filter question for some of the following items).

S6 Script. Assumption testing. This R script will be used for testing the assumptions of the statistical models used to test hypotheses 1 and 2.

S7 Script. Preliminary analysis script. This script will be used to analyze the data of the survey. It includes all steps of preprocessing, as well as descriptive and confirmatory analyses. If necessary, small changes may be made to enable correct functioning (when variables have been inserted). Furthermore, analyses might be added if alternative approaches need to be used (due to assumption violation).

Click here to access/download;Figure;Fig1.tif

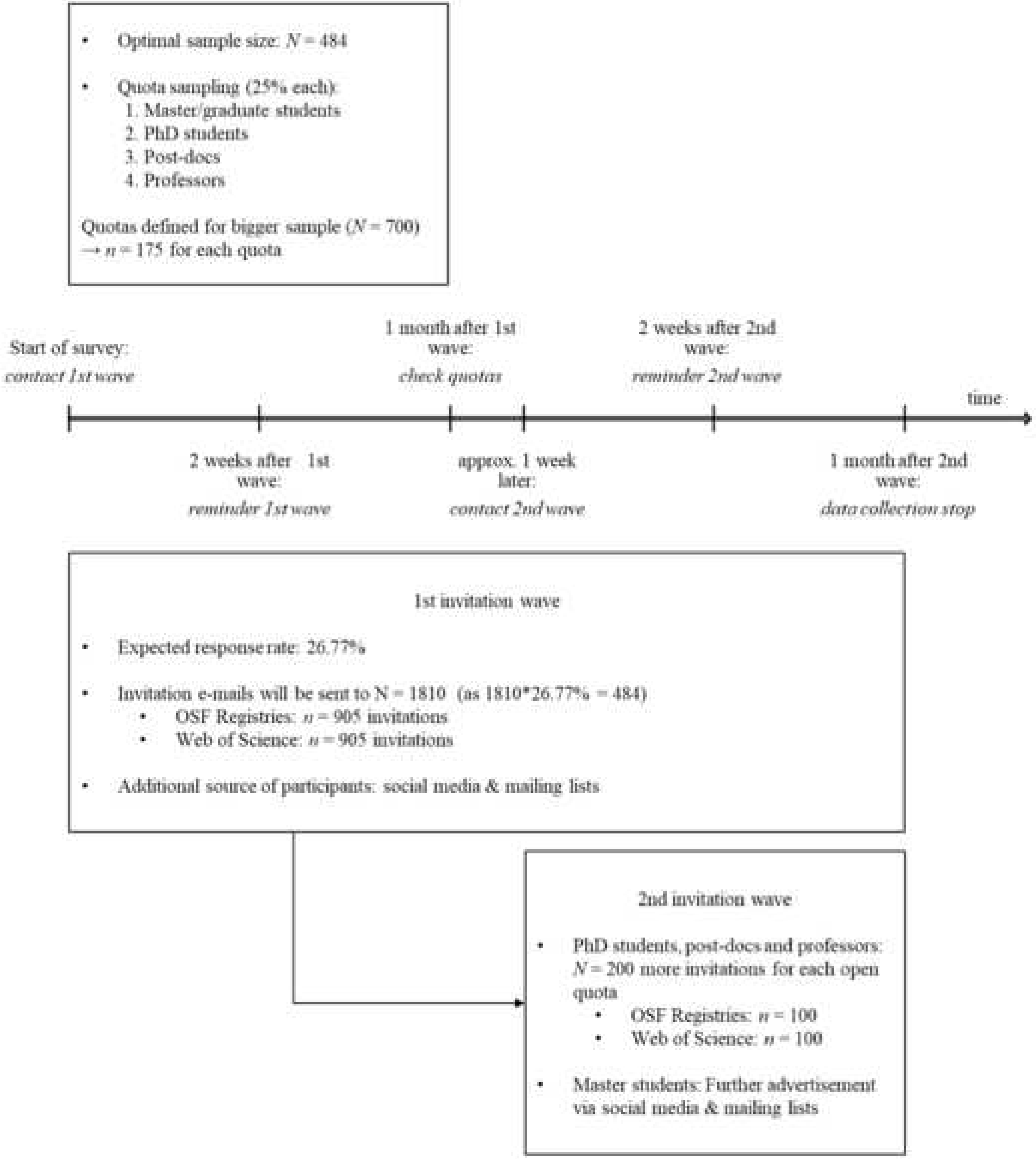

Power analyses

Click here to access/download
Supporting Information
S1_Text.docx

Detailed data collection procedure

Click here to access/download
Supporting Information
S2_Text.docx

Survey items

Click here to access/download
Supporting Information
S3_Table.xlsx

Screen recording of survey (version A)

Click here to access/download

Supporting Information

S4_Video.mp4

Screen recording of survey (version B)

Click here to access/download
Supporting Information
S5_Video.mp4

Script for assumption testing

Click here to access/download
Supporting Information
S6_Script.R

Analysis script

Click here to access/download
Supporting Information
S7_Script.R
