# Supplementary material for: Registered Report Protocol: Survey on attitudes and experiences regarding preregistration in psychological research
Source: PLoS One. 2021 Jul 2;16(7):e0253950. doi: 10.1371/journal.pone.0253950 (PMC8253437; doi:10.1371/journal.pone.0253950)
Supplement: Attachment — Submitted filename: renamed_5f626.docx [file pone.0253950.s009.docx]

**COMMENTS – Manuscript PONE-D-20-20445**

Title: “Attitudes and experiences regarding preregistration in psychological research”

**Important note: This review pertains only to ‘statistical aspects’ of the study and so** **‘****clinical aspects’ [like medical importance, relevance of the study,** **‘clinical significance and implication(s)’ of the whole study, etc.] are to be evaluated [should be assessed] separately/independently. Further please note that any ‘statistical review’ is generally done under the assumption that (such) study specific methodological [as well as execution] issues are perfectly taken care of by the investigator(s). This review is not an exception to that and so does not cover clinical aspects {however, seldom comments are made only if those issues are intimately / scientifically related & intermingle with ‘statistical aspects’ of the study}. Agreed that ‘statistical methods’ are used as just tools here, however, they are vital part of methodology [and so should be given due importance].**

**COMMENTS:** Your ABSTRACT is well drafted but assay type. Because your article type is ‘Registered Report Protocol’ on a very special viewpoint on preregistration of studies, I can understand that it is difficult to covert it as ‘Structured summary’. However, it will definitely be more informative if you can do that, I guess [even if your article type is ‘Registered Report Protocol’]. Please note that it is preferable to divide the ABSTRACT with small sections like ‘Objective(s)’, ‘Methods’, ‘Results’, ‘Conclusions’, etc. which is an accepted practice of most good/standard journals [including PLOS] whatever the article type may be.

Why it is not indicated in the title that this a ‘Protocol’? [in my opinion, it is better as it helps reader(s) to read the article with that perspective].

In the context of material given in lines 290-3, it may be noted that

“Whenever response options ranged from 1=strongly disagree to 4=strongly agree (or from 1=very bad to 3=neither good nor bad to 5=very good), while using a ‘Likert’ scale responses, recoding [like strongly disagree=-2, disagree=-1, neutral=0, agree=1, strongly agree=2] may yield correct and meaningful ‘arithmetic mean’ which is useful not only for comparison but has absolute meaning, in my opinion. Application of any statistical test(s) assume that meaning of entity used (mean, SD, etc) has a particular meaning. Though ‘α’ [alpha] or most other measures of reliability/correlation will remain same, however. Use of non-parametric methods should/may be preferred while dealing with data yielded by any questionnaire/score.”.

Where and how to use it will be decided by the authors. But it is given here because I thought that it may be useful [for all analyses (including ‘Confirmatory analyses’ described in lines 375-437)] proposed in this protocol. Though I am not familiar with most of the ‘software’(s) named in ‘Data analysis’ section [lines 375-429], I am confident that authors will use them appropriately.

Because the topic of this study is very interesting as well as going to be very useful, in my opinion, worth publishing (may be after minor revision).
