# Supplementary material for: Registered Report Protocol: Survey on attitudes and experiences regarding preregistration in psychological research
Source: PLoS One. 2021 Jul 2;16(7):e0253950. doi: 10.1371/journal.pone.0253950 (PMC8253437; doi:10.1371/journal.pone.0253950)
Supplement: Attachment — Submitted filename: Peer Review Registered Report Spitzer-2.docx [file pone.0253950.s010.docx]

The reviewer wants to congratulate the authors on this excellent registered report.

Not only is the study itself important and can give a clearer picture of pre-registration, the fact that the authors are completely open with their materials is highly appreciated. Furthermore, the authors are guiding the reader easily and clearly through their methods.

Especially the videos, guiding through the survey, show high transparency.

However, some points still need to be clarified. Thus, I give the following recommendation.

*Revision with minor changes*

Minor remarks:

Title: needs to be clearer. No word of survey although this represents the heart piece of the study, registered report, quality of research

Abstract:

L 15 Immutable. Really? It is mutable and at least in OSF these dates can be tracked (you state so yourself in l 140)

L 109: More explanation about Registered Report is missing especially as this manuscript is one itself. The explanation seems too short, when there is a long explanation of pre-registration. Registered Reports are a new form of pre-registration and include peer review whereas pre-registrations don’t. Moreover, preregistration is a faster way to publish, as it has to go through no quality check.

L 150: Wording. If you want to keep this phrase, please include “to our knowledge”

L 186 How will you cope with the different age of professors? ERC / Young professors might be more open to the idea of Pre reg than ones who are close to retirement.

L 233: Will the search be executed by two people ?

L 233 Don’t you introduce a huge bias if you select half of the participants from OSF. I see the discussion in L 401 but still would like to have more explanation

L 242: please put the calculation in the extra file and only put the sources, or give the min and max values and only put the references

L 243: For the value 26.77 you put two numbers after the decimal point and this seems strange as before you always went with one. However, probably this format has been chosen to not change the sample size of 484.

L 268: Is excluding Master students who will not continue in Research not introducing a bias? How do you make sure the ones who say they stay in academia the ones who will leave do so they because of the lack of pre-registration and open science in their lab?

L 276 The survey language is English, or are there other options as well (German)?

Major remarks

Title:

Please give more information about what kind of research this is (survey + registered report)

Abstract:

I know that there is no specific guideline for registered reports. However, try to stick to one. Like PRISMA or STROBE.

Please write more about the methods you will use. I am missing the searched databases OSF/Web of science and that this includes an online survey.

No words about the different groups studies (PhD, Postdoc, ...) could be found.

Furthermore, the number of subjects studies is missing.

General comments:

Adding a section of Limits / Risk would be good

(e.g. is external validity given, if half of sample size already registered a protocol)

What if, if still after two rounds the quotas for professors might not be filled?

Or what if you find 175 professors who are willing to participate but not all of them will finish the study.

What to do in case of deviations or if too many results show ceiling or floor effects.

What is the time frame of the study? Can this study be finished in one year?

I declare myself unable to assess the statistical methods used.
