# Supplementary material for: Registered Report Protocol: Survey on attitudes and experiences regarding preregistration in psychological research
Source: PLoS One. 2021 Jul 2;16(7):e0253950. doi: 10.1371/journal.pone.0253950 (PMC8253437; doi:10.1371/journal.pone.0253950)
Supplement: Attachment — Submitted filename: plos-review-kd.docx [file pone.0253950.s012.docx]

**Summary**

You plan to survey psychology researchers and students who regularly publish preregistrations on OSF registries, author Web of Science articles, or use social media or mailing lists. The survey will test the primary hypothesis that attitudes, norms and perceived control predict intention to preregister, and the secondary hypothesis that attitudes, motivations and perceived obstacles differ between academic groups. You informed your hypotheses and survey measures with the theory of planned behaviour.

**Comments**

Firstly, the detail of your study is impressive. A lot of your method is clearly described and the summative figures, videos, and tables you provided help to digest it. Your stopping rule and adjustments for low response rates seem well thought out and justfied, as does your use of theory. I was also very pleased that you cited all the R packages you used.

Below I outline some minor and major comments I have on the design and reporting of your study. I discussed my comments with one other reviewer of this paper and other people from my research group so some may appear similar.

**Minor points**

- I think you undersell the role of other fields in open science. Preregistration, preprints and data sharing have a longer history and larger uptake in other fields. This doesn't diminish the importance of preregistration in psychology but it does suggest that psychology is not the only driving force for open science.
- You use brackets frequently and I sometimes found it quite difficult to read. May be worth moving some of the bracketed content into a full sentence to make it more readable.
- I suggest adding a "prefer not to answer" option to the survey's gender question. This will allow respondents protect their identity and may avoid some people dropping out.
- I think your introduction is too long and duplicates existing review papers on the open science movement. Signposting readers to an appropriate open science review article could help cut your introduction down.
- You sometimes cite opinion articles as evidence. For example "Preregistration also prioritizes theorizing and methods over results as the main criterion for judging the quality of research [20]". van't Veer and Giner-Sorolla may have this priority but I do not know how many people who pre-register do. You also say "Analytical flexibility is decreased by defining and documenting planned analyses beforehand because this way one can clearly distinguish confirmatory from exploratory analyses [9,11,20,29]." You cite four opinion articles so again this is a belief of the authors not a fact. For statements like these, I would cite an empirical article supporting your statement (e.g. an article comparing outcome switching in pre-registered and unregistered clinical trials), remove it or qualify it.
- I am unsure how you are using the terms "systematic evaluation focusing on preregistration" and "QRPs". Definitions would help.

**Major points**

- I am nervous about how you will detect and control for possible selection bias from advertising the study on social media. Will you be collecting the recruitment method for each participant? If yes, will you examine and control for any differences between recruitment sources? How?
- The development and validity of your measures is unclear. You do not give existing validity evidence for measures you adapted from other survey. For the measures you created yourself, the validity evidence is unclear as is your rationale for the response options for these measures (for example why did you use 7-point scales for some and 4-point scales for others). To me, a more transparent description of your measures would allow others to replicate and evaluate them. Flake and Fried (2019, January 17) provide a list of questions to promote transparency in measurement that may be useful (https://psyarxiv.com/hs7wm/).
- I saw no reference to any piloting of the survey. If you have not piloted I recommend you do to understand, for example, when and why people drop out, if the survey is appropriate for people across different countries or non-native English speakers, and if people interpret the questions in the way you expect them to.
- How will you use data from the question "How would you define the term "preregistration"?". You do not seem to plan to screen out people who understand preregistration differently to the open science definition but I recommend you do this. For example, you could describing preregistration at the start and include a "knowledge check" to ensure respondents are understanding it correctly.
- Please be specific about what you powered your study to detect in the main body of the article. On line 200 you do not say what effect or effect size you have 95% statistical power to detect.
- Your qualitative analyses are not described in enough detail for me to replicate them. To ensure others can replicate your analyses you can describe how you will analyse words quantitatively

I hope you find my feedback and suggestions useful.

Katie Drax
