# Supplementary material for: Registered Report Protocol: Survey on attitudes and experiences regarding preregistration in psychological research
Source: PLoS One. 2021 Jul 2;16(7):e0253950. doi: 10.1371/journal.pone.0253950 (PMC8253437; doi:10.1371/journal.pone.0253950)
Supplement: Attachment — Submitted filename: pone-review2-kd.docx [file pone.0253950.s014.docx]

**Comments**

It was a pleasure reviewing this again. You have done an admirable job responding to my original comments and I’m glad that the pilot study was useful for you. Overall, I think your responses are excellent in that they have provided more clarity and justification for your proposed method.

Below I have outlined my thoughts on points I brought up in the previous review cycle and new issues.

**Previous comments**

- You have resolved all my previous minor points and my previous major points about piloting, the power analyses, and qualitative analysis.
- Lines 244-249 imply to me that “a sample biased toward researchers who have already preregistered… is not an issue.. on identifying problems in the current implementation of preregistration and uncovering reasons for psychological researchers’ potential ambivalence toward it.”. I agree a biased sample may help identify some problems and reasons but only the ones relevant to the sample. We will not know if or how they exist in the wider population. A major issue in the sample may only be minor in wider population. Reframing these lines as a limitation would be clearer.
- I do not understand the rationale for the numbers of items or the process by which you came to that number (24 items for attitudes, 1 for perceived importance, 8 for subjective norm, etc). Making this clearer will help readers understand the justification for the measures and the survey length.
- It is unclear to me how you chose the options to the non-scale items (e.g. drawbacks of preregistration). Providing a rationale for them would allow readers to replicate and evaluate the measures. The options could also be quite leading. Changing all such items to open text and analysing them qualitatively would reduce the risk of options being leading.

**New comments – minor points**

- Line 52 - I would change this to “Preregistration – on the rise?”
- I didn’t see an explanation of how you will identify and exclude “bot” responses. Including a statement about this would make this clear – see this tweet for further details <https://twitter.com/m_simonephd/status/1174010078632009728>
- I would probably fail the “knowledge check” because I would not check the box that “preregistration will increase transparency”. I believe preregistration could increase transparency, but that this is not a guarantee. “Bad” and poorly detailed preregistrations are possible. Removing the “increases transparency” check from the knowledge check question would avoid people like me failing the check.
- If I was a participant, I would find it helpful to have labels above each of the 7-point items (e.g. strongly disagree – disagree- somewhat disagree – neither agree nor disagree – somewhat agree – agree – strongly agree). Otherwise, I would not be sure what the “neutral” answer was, or I’d have to count along the row to find the neutral answer.
- Sensitivity analysis to examine how your exclusions affect the results could be useful
- Lines 277-281. It is unclear to me how excluding masters students who do not intend to pursue an academic career will mean that your survey will target a “research-oriented psychology” population. What about members of the other groups who intend to leave academia very soon (especially relevant for PhD students)? The question also means you run the risk of excluding master’s students who don’t intend to go into academia but ultimately do, and including those who do intend to continue but don’t. I recommend you clarify why you are not excluding other groups who intend to leave academia soon and how you will handle the risk of master’s students’ intentions not matching their behaviour. Alternatively, you could ask members of all groups if they intend to pursue an academic career and conduct sensitivity analyses to see if differences occur based on this intention.

**New comments – major points**

- The introduction contains little discussion of relevant theories and their respective strengths and limitations. I think there has been lively debate around the utility and validity of the theory of planned behaviour (e.g. <https://www.tandfonline.com/doi/full/10.1080/17437199.2013.869710>). I would understand the theoretical basis of your survey better if you explained why you chose the theory of planned behaviour over others, if the debates about the theory of planned behaviour apply and how you will handle them.
- Measurement validity remains a key concern for me but for more reasons than before. I’m glad you found the Flake & Fried paper useful and lines 317-320 now make the validity of the survey clear. The lack of validity measures mean I feel I do not have evidence that your measures are measuring what you intend them to. I believe this will weaken the evidence your study can provide but I understand this may be an unavoidable limitation.

I understand that me bring up new issues on the second round may be difficult but I hope they are still useful.

Kate Drax
