# Supplementary material for: Registered Report Protocol: Survey on attitudes and experiences regarding preregistration in psychological research
Source: PLoS One. 2021 Jul 2;16(7):e0253950. doi: 10.1371/journal.pone.0253950 (PMC8253437; doi:10.1371/journal.pone.0253950)
Supplement: Attachment — Submitted filename: Response to reviewers.docx [file pone.0253950.s015.docx]

***Response to reviewers***

We thank the reviewers for reading our revised manuscript thoroughly and for providing constructive criticism. We believe that by addressing all points to our best knowledge, we were able to improve the study further, and we hope that you now feel that the revised manuscript can be recommended for an in-principle-acceptance.

Overall, we have implemented various strategies to counter a potential sampling bias by adjusting both our recruitment strategy and our analyses. We further updated our exclusion criteria and added a sensitivity analysis. Furthermore, we decided to change our approach toward the distinction of early career vs. senior researchers by rather using the more continuous variable of research experience, i.e., the number of years someone has engaged in psychological research. Accordingly, we will compute regression models investigating attitudes and the perceived strength of motivations and obstacles (hypothesis 2) rather than a MANOVA. Since research experience is measured by a new item that was not included in the previous version of the survey, we will not use the pilot data for the main study.

More details about the changes are included in our responses to each reviewers’ comments below. If line numbers were given by the reviewers, we include the term, sentence, or section, to which the comment refers to, in parentheses.

***Reviewer #1***

*Since most of the comments made on earlier draft by me (and hopefully by other respected reviewers also) are attended positively/adequately, I am fully satisfied and the manuscript is improved a lot.*

*Particularly, what you said (while answering my comment) [When the Stage 2 Registered Report is submitted, the sections “results” and “discussion” will be added and we may move confirmed hypotheses to the results section] is perfectly right/ appropriate. Change made in ‘title’ [“Registered Report Protocol: Survey on ……] is highly appreciated. Changes made in the “scales” also are appreciable/very good.*

We are very pleased that you feel we have improved the manuscript and that you are now fully satisfied with it. Thank you very much for your constructive comments, which helped us a lot in the revision process.

***Reviewer #2***

*I thank the authors for addressing all points. I know how much time it has taken, especially in the case of a registered report this can be agonizing. However, this project is a nice piece of research and I am looking forward to the results.*

We would like to thank you for your valuable assistance with our study. We are very pleased that you find our research interesting and we are also looking forward to the results.

***Reviewer #3***

*The revised Stage 1 RR manuscript has been improved in many ways. I applaud the authors for their thorough work. There remain, however, at least two major concerns.*

*1. Sampling bias. The authors state that they are not concerned about sampling bias:*

*“this is not an issue for our survey because the main focus does not lie on gaining a representative sample of all psychological researchers, but on identifying problems in the current implementation of preregistration and uncovering reasons for psychological researchers’ potential ambivalence toward it.”*

*Based on their sampling plan, the sample will be heavily biased towards researchers who have already preregistered and are familiar with it. 50% of their sample will come from OSF registries, and thus by definition have already preregistered, and 90% of their pilot sample have experience with preregistration. Thus, their sentence above would need to be modified to state: “…uncovering reasons for psychological researchers’ [who have already used preregistration] potential ambivalence toward it.” I find that clearly stating this limited generalizability due to the selective/biased sample makes the question seem much less interesting. Similarly, the hypotheses become much less interesting (e.g., “1.1. [Of researchers who are familiar with preregistration,] More beneficial attitudes are a positive predictor for the intention to preregister”. “2.2. Early career researchers (PhD students) [who have used preregistration] differ from senior researchers (postdocs, professors) [who have used preregistration] in their attitudes (i.e., how positive they view preregistration).”*

*All this to say, the study could be run with a sample of participants that are familiar with preregistration, but all the conclusion would need to be tempered to ensure that they don’t make claims beyond the selective sample. I know PLOS ONE focuses on ensuring that the methods are sounds and is largely agnostic to the importance of research, but I am concerned that this sampling bias would inhibit the researcher from answering the question they are interested in answering. I recommend aiming for a representative sample of psychology researchers. This could be done by using Web of Science, Scopus, and other databases (as a side note, PubMed may lead to a sample of psychology researcher more concerned with the medical side of psychology).*

*If the authors decide to continue to sample from the OSF REGISTRIES, I strongly believe that this sample should be analyzed separately from those from the Web of Science and PubMed samples, because they are drawn from what I would consider different populations. Thus, summary statistics that combine these samples don’t represent a real population.*

Thank you for your thorough feedback. We have thought deeply about this criticism and think that it is very helpful that you addressed and highlighted it. To counter this bias, we will use the following strategies:

We will increase recruiting through general databases to achieve a sample that resembles the real population of psychological researchers better. We consulted one of our in-house experts from the department of information services of our institute for this purpose and decided to use the following databases: Web of Science, PubMed, PSYNDEX, and PsycInfo. Through each of these databases, 22.5% of participants will be invited (90% in total). Meanwhile, the percentage invited through OSF will be significantly reduced to 10% instead of 50%. Of course, recruitment via OSF could be eliminated altogether, however, Hardwicke et al. (2020) have shown that it might be the case that only very few studies are preregistered. So it could be that in the general sample hardly any researchers preregistered. Since we are also interested in the experiences of researchers who prepared and submitted a preregistration themselves, we decided to still invite a small fraction of the sample via OSF (10%) to ensure a subsample with preregistration experience. We have described this in detail in the section *data collection and stopping rule* (p. 12 ff).

Nevertheless, this recruitment strategy might still lead to a bias. We will control for this in the following way: We will make the general statement of what proportion of researchers have already preregistered only for the sample recruited via the general databases (i.e., we achieve a representative statement here, as we exclude the “biased” OSF sample). We will run all further descriptive reports separately for researchers who have preregistered before vs. not and compare between these groups (see section *descriptive reports*, p. 20 ff). We also want to account for possible biases in our hypotheses tests and therefore decided to include “preregistration experience” as a dichotomous control variable (see section *hypotheses tests*, p. 22 ff).

By including these various control strategies, we think that we now control well for the potential sampling bias.

*2. Sampling plan and pilot study. The authors calculate their sampling plan with a 10% response rate from the pilot study. However, there were no Master’s students or postdocs who completed the survey, and thus the response rate is 0% for these groups. The authors do not report how many participants in the pilot were from the 3 samples of OSF, Web of Science, and PubMed. For their sampling plan to work as hoped, the response rate would need to be 10% from each of these platforms in each of the 4 career stages, which their data show is not the case. Furthermore, it is unclear how additional participants from social media and mailing lists will be used in the analysis. These additional participants introduce an additional sampling bias.*

We conducted the pilot study because we wanted to know more about the overall response rate and test the recruitment method and the survey items. We don’t want to draw conclusions about more fine-grained distributions such as the return rate for different groups or databases based on this small sample (we also deleted this information from the manuscript as it might be misleading). However, our recruitment strategy anticipates that different academic groups will not be equally represented. To compensate this, we set quota variables for different academic degrees and intend to explicitly recruit participants for the open quotas in the second wave of invitations (see Fig 2).

Regarding the databases, we do not employ quotas as we believe that participants whose publications appear on the general databases (Web of Science, PubMed, PSYNDEX, PsycInfo) will overlap (though duplicates will be excluded) and we have no reason to believe that their attitudes toward preregistration or their response rate differ depending on the database. This is different for participants recruited from OSF Registries. Here we suppose that attitudes toward preregistration will be more positive as these participants were identified based on their preregistration. To counter this bias, we will include preregistration experience in the respective analyses as outlined above. This may also help, at least partly, in terms of a self-selection bias, that is, that people who are inherently more interested in preregistration may be more likely to participate in the survey. This could also apply to people who were recruited from social media. Additionally, we will distribute different survey links per database and for social media posts which will allow us to infer the recruitment source and thus, to check post-hoc for differences.

*Minor comments:*

*1. Line 64. Preregistration is mandated in medicine, although I’m not sure if it’s the norm. Many studies are still not registered or they are registered retrospectively.*

*[L 64 ff: However, while preregistration is already the norm in other scientific disciplines like medicine [18], it has been frequently demanded as a means to counter questionable research practices but is still not widely practiced in psychology.]*

We have changed the sentence to “However, while preregistration is already well-established in other scientific disciplines and is mandated, for example, in medicine [18], it has been frequently demanded as a means to counter questionable research practices but is still not widely practiced in psychology.” (p. 4, l. 64 ff).

*2. Explain how scores such as “beneficial attitudes” will be calculated. Will it just be the mean of all those survey questions?*

Yes, we will calculate the attitude score by computing the mean for each participant over all attitude items (24 items). The same procedure will be done for the subjective norm scale (mean of eight subjective norm items), the perceived behavioral control scale (mean of five perceived behavioral control items), the intention scale (mean of three intention items), the motivation scale (mean of ten motivation items), and the obstacle scale (mean of ten obstacle items). We made this clearer in the manuscript in the section *material* (p. 15 ff): “For the statistical analyses, the mean scores of the scales will be used to measure how participants perceive 1) preregistration (attitude scale), 2) subjective norms regarding preregistration (subjective norm scale), 3) their own control about using preregistration or not (behavioral control scale), 4) their intention to use preregistration in the future (intention scale), and 5) their motivations (motivation scale) and 6) obstacles to preregister (obstacle scale). For each participant, the mean for each scale will be calculated and used as the score.” (p. 17, l. 338 ff).

*3. Your specific usage of the term Early Career Research will confuse readers as this term almost always encompasses postdocs. I recommend simply saying PhD students.*

We are very thankful for this comment, and have thought about it in depth. We have concluded that our classification into academic groups was ambiguous and the distinction between early career researches and senior researchers unnatural since experience may be better represented on a more continuous scale. Therefore, we decided to change the operationalization of research experience into a more objective one: We will use the highest academic degree in psychology for the descriptive reports, and define research experience by the years someone has already worked in psychological research.

Hypothesis 2 will consequently be tested via multiple regressions with research experience as a predictor for 1) attitudes, 2) motivations, and 3) perceived intensity of obstacles (see section *hypotheses tests*, p. 22 ff). These will replace the previously planned MANOVA.

The preregistration experience will be included as a control variable. We hope to achieve more meaningful conclusions by using a more objective, clear, cross-regionally transferable, and continuous variable as predictor for our scales.

*4. Will your study include research associates, readers and lecturers (i.e., non-full professors)?*

We have decided against using our previous classification altogether (see our comment above). For the descriptive reports, we will group participants based on their degree (bachelor's degree, master's degree, doctoral degree, habilitation or full professorship), and for our hypotheses tests, we will use research experience defined as the years someone has already worked in psychological research.

*On another note, I think that the survey responses presented as descriptive statistics would be interesting even if the sample was biased.*

*In summary, I believe (1) the sampling bias needs to be minimized, or the authors should present a strong argument for why sampling bias is not a concern (which I don’t believe has been presented so far). And (2) that evidence is needed that the sampling plan will be sufficient to achieve the desired numbers in each category; perhaps demonstrable with a larger pilot and revised categories or sample size.*

*I am sympathetic to research on preregistration and also conduct some with my group. I hope these comments are taken in the collegial manner intended.*

*I always sign my reviews,*

*Robert Thibault*

Once again, we would like to thank you for the extensive work you have put into your reviews of our study so far. We have thought thoroughly about your comments and tried to address them to our best knowledge. We hope that you now find our recruitment strategy and categories more appropriate, and are looking forward to your feedback.

***Reviewer #4***

*I responded “Partly” to questions 1 and 2 because the validity of the measures in the survey remain a key concern for me. I would also like to know more about the theoretical basis of the study. See the attached comments document for more information.*

*It was a pleasure reviewing this again. You have done an admirable job responding to my original comments and I’m glad that the pilot study was useful for you. Overall, I think your responses are excellent in that they have provided more clarity and justification for your proposed method.*

*Below I have outlined my thoughts on points I brought up in the previous review cycle and new issues.*

Thank you very much for your previous comments and for your feedback. We have tried to address your comments in detail, as described below.

*Previous comments*

*You have resolved all my previous minor points and my previous major points about piloting, the power analyses, and qualitative analysis.*

We are happy that you are satisfied with our implementation of your previous comments.

*Lines 244-249 imply to me that “a sample biased toward researchers who have already preregistered… is not an issue.. on identifying problems in the current implementation of preregistration and uncovering reasons for psychological researchers’ potential ambivalence toward it.”. I agree a biased sample may help identify some problems and reasons but only the ones relevant to the sample. We will not know if or how they exist in the wider population. A major issue in the sample may only be minor in wider population. Reframing these lines as a limitation would be clearer.*

*[L 244 ff: This recruitment procedure might result in a sample biased toward researchers who have already preregistered (see section pilot study). However, this is not an issue for our survey because the main focus does not lie on gaining a representative sample of all psychological researchers, but on identifying problems in the current implementation of preregistration and uncovering reasons for psychological researchers’ potential ambivalence toward it.]*

We agree and since we indeed want to, at least partly, make statements about the wider population of psychological researchers, we decided to more deliberately protect against this sampling bias. In particular, we amended our recruitment strategy by including more general databases and by inviting less participants via OSF Registries. To examine the percentage of preregistrations in the wider population, the subsample from OSF Registries will be excluded or reported separately. When testing our planned hypotheses, we will include preregistration experience as a control variable (please see our detailed response to a similar comment by reviewer #3 above). By using these new approaches, we hope to be able to make statements about a larger part of the psychological research community. Persisting limitations will be addressed when discussing the results.

*I do not understand the rationale for the numbers of items or the process by which you came to that number (24 items for attitudes, 1 for perceived importance, 8 for subjective norm, etc). Making this clearer will help readers understand the justification for the measures and the survey length.*

For our scales, we took a content-guided approach. Therefore, the number of items is determined by how many different aspects a scale covers. Since we assume, for example, that attitudes are a very complex construct, we have included a larger number of items for this scale in order to be able to represent this complexity. Meanwhile, the motivation and obstacle scale have the same length (ten items) to evenly display motivations and obstacles. Lastly, the number of items of the subjective norm scale (eight items) and the perceived behavioral control scale (five items) was chosen to reflect some of the most important aspects of these constructs, without targeting a specific number of items.

Following your comment, we have studied a manual for constructing TPB questionnaires to ensure that our design is appropriate. Based on the manual (“Constructing questionnaires based on the theory of planned behaviour: A manual for health services researchers”, Francis et al., 2004), we have added two questions inquiring about the intention to use preregistration in the future. We will now use the mean score of this intention scale as dependent variable for hypothesis 1 (rather than the response to the single item that we had before). Our other items were already in accordance with the guide. We hope that using this guide further improves the validity of our questionnaire.

*It is unclear to me how you chose the options to the non-scale items (e.g. drawbacks of preregistration). Providing a rationale for them would allow readers to replicate and evaluate the measures. The options could also be quite leading. Changing all such items to open text* and analysing them qualitatively would reduce the risk of options being leading.

Our initial idea was to reduce the effort for the participants and obtain a ranked order of the options that were selected based on literature research and personal experience. However, we agree that providing options may limit the responses of participants and thus decided to retract to open text inputs as suggested.

Specifically, given answer options were deleted for the items:

1) What are positive/negative consequences of mandatory preregistration?

2) Benefits of preregistration

3) Drawbacks of preregistration

4) Reasons against preregistration

Nevertheless, we would like to keep multiple choices for the two items addressing problems and worries in the context of preregistration (i.e., the questions “Did you encounter specific problems when preregistering a study? If yes, which ones?” and “What worries do you have with respect to preregistering your studies?”) as they are matched. That is, the same aspects are mentioned in both items but the items are presented to different groups: Researchers who have not yet preregistered are asked about their worries and researchers who have preregistered their studies in the past are asked about the problems that they encountered. This matching makes it possible to specifically compare worries and experienced problems.

*New comments – minor points*

*Line 52 - I would change this to “Preregistration – on the rise?”*

*[L 52: Preregistration – At the rise in psychology?]*

We have updated the section title accordingly (cf. p. 4, l. 52).

*I didn’t see an explanation of how you will identify and exclude “bot” responses. Including a statement about this would make this clear – see this tweet for further details https://twitter.com/m_simonephd/status/1174010078632009728*

We included a captcha question at the start of the survey (i.e., an arithmetic task, cf. p. 18, l. 352 f; we decided against using the recaptcha plugin by Google due to privacy concerns). Furthermore, we will distribute different links to researchers recruited via the different databases (i.e., OSF Registries, Web of Science, PubMed, PSYNDEX, PsycInfo) and social media.

*I would probably fail the “knowledge check” because I would not check the box that “preregistration will increase transparency”. I believe preregistration could increase transparency, but that this is not a guarantee. “Bad” and poorly detailed preregistrations are possible. Removing the “increases transparency” check from the knowledge check question would avoid people like me failing the check.*

We changed the option to “Preregistration aims at increasing the transparency of potential changes made to a study.” to reduce ambiguity.

*If I was a participant, I would find it helpful to have labels above each of the 7-point items (e.g. strongly disagree – disagree- somewhat disagree – neither agree nor disagree – somewhat agree – agree – strongly agree). Otherwise, I would not be sure what the “neutral” answer was, or I’d have to count along the row to find the neutral answer.*

For all scale items, the suggested labels were included (however, “slightly” was used instead of “somewhat”). The labels included now are: “strongly disagree - disagree - slightly disagree - neither agree nor disagree - slightly agree - agree - strongly agree” (also see S4 and S5 Videos).

*Sensitivity analysis to examine how your exclusions affect the results could be useful*

All participants that indicate that their research or studies do not fall within the scope of psychology or that do not have at least a bachelor’s degree in psychology, and thus, cannot be assigned to the respective quota, will be screened out at the beginning of the survey, thus, in this case, no sensitivity analysis is possible. However, for our other exclusion criteria, we will conduct sensitivity tests. Specifically, we will repeat the hypotheses tests while also including participants that have not completed all pages of the survey (see section *exclusion and missing data*, p. 14 f).

*Lines 277-281. It is unclear to me how excluding masters students who do not intend to pursue an academic career will mean that your survey will target a “research-oriented psychology” population. What about members of the other groups who intend to leave academia very soon (especially relevant for PhD students)? The question also means you run the risk of excluding master’s students who don’t intend to go into academia but ultimately do, and including those who do intend to continue but don’t. I recommend you clarify why you are not excluding other groups who intend to leave academia soon and how you will handle the risk of master’s students’ intentions not matching their behaviour. Alternatively, you could ask members of all groups if they intend to pursue an academic career and conduct sensitivity analyses to see if differences occur based on this intention.*

*[L 275 ff: All participants that indicate 1) that their research does not fall within the scope of psychology (or for master students, whose major is not psychology), 2) that they are neither a master student, PhD student, postdoc nor professor, or 3) that they are master students who do not intend pursuing an academic career, will be screened out at the beginning of the survey. Thus, they will be directed to an exit page rather than to the main body of the survey, will not be counted into the quotas, and will not be considered for data analyses as the survey targets a sample from research-oriented psychology.]*

As suggested, we will include all psychologists with at least a bachelor’s degree (formerly the group of master students) and present the question: “Do you plan to continue your academic career, or leave academia? If you are unsure, please indicate your current tendency here.” to all participants for possible post-hoc analyses.

*New comments – major points*

*The introduction contains little discussion of relevant theories and their respective strengths and limitations. I think there has been lively debate around the utility and validity of the theory of planned behaviour (e.g. https://www.tandfonline.com/doi/full/10.1080/17437199.2013.869710). I would understand the theoretical basis of your survey better if you explained why you chose the theory of planned behaviour over others, if the debates about the theory of planned behaviour apply and how you will handle them.*

Thank you for pointing this out. We decided to use the TPB as the basis for our first hypothesis since it is still widespread, relevant, and promising today. For example, it has been argued to be the most widely used theory in the field of human decision research (e.g., see “The Enduring Use of the Theory of Planned Behavior”, Miller, 2017). Supporting this claim, a large pool of studies has addressed the theory of planned behavior, as demonstrated by a search on Web of Science showing that 2029 articles which contain the keywords “theory of planned behavior” have been published in the last five years alone. Furthermore, it has been tested numerous times by meta-analytical approaches with promising results (e.g., see [40–45] of our manuscript).

We will acknowledge potential shortcomings of the theory in the following way: While its flexibility is often pointed out as a virtue of the theory, the parsimony of its variables is also criticized. We will extend the theory by adding a moderating variable (“perceived importance of preregistration”) as well as a control variable (“preregistration experience”) to make better predictions. Another criticism is that the theory is not falsifiable, especially since in the case of null effects researchers oftentimes criticize their study design rather than the theory. Our study is a Registered Report and is reviewed and revised for methodological rigor before it is conducted. We will take this into consideration and be careful to not interpret null effects as design flaws only, but such that the theory might not be able to predict intentions in our context. Other concerns were voiced regarding validity and utility of the model. In this regard, we refer to the meta-analyses pointed out in the manuscript (cf. p. 7, l. 127) which we believe highlight the benefits of the theory.

We have added a summary of our arguments for using the theory in the manuscript (cf. p. 6, l. 115 ff).

*Measurement validity remains a key concern for me but for more reasons than before. I’m glad you found the Flake & Fried paper useful and lines 317-320 now make the validity of the survey clear. The lack of validity measures mean I feel I do not have evidence that your measures are measuring what you intend them to. I believe this will weaken the evidence your study can provide but I understand this may be an unavoidable limitation.*

Thank you for pointing this out. We fully agree with your concern, and we will mention this limitation in the discussion section of our Registered Report Article.

*I understand that me bring up new issues on the second round may be difficult but I hope they are still useful.*

*Kate Drax*

Thank you for both your feedback to your previous comments and your new comments as we feel that your suggestions further improved our planned study. We are now looking forward to your feedback.
