# Supplementary material for: Registered Report Protocol: Survey on attitudes and experiences regarding preregistration in psychological research
Source: PLoS One. 2021 Jul 2;16(7):e0253950. doi: 10.1371/journal.pone.0253950 (PMC8253437; doi:10.1371/journal.pone.0253950)
Supplement: Attachment — Submitted filename: Response to reviewers.docx [file pone.0253950.s016.docx]

***Response to reviewers***

We thank the reviewers for reading our revised manuscript thoroughly and for providing constructive comments and criticism. We believe that by addressing all points to our best knowledge, we were able to improve the study further, and we hope that you now feel that the revised manuscript can be recommended for an in-principle-acceptance.

Overall, we have further improved our recruitment strategy to ensure that we will reach our targeted sample size, made small changes to the survey to improve its comprehensibility, and added some clarifying descriptions to our manuscript. Furthermore, we reviewed our reference list.

More details about the changes are included in our responses to each reviewers’ comments below. If line numbers were given by the reviewers, we include the term, sentence, or section, to which the comment refers to, in parentheses.

***Reviewer #3***

*The authors addressed my main concern about sampling bias. My concern about achieving their sample size remains. Based on their pilot data, and my best guess, they will need to invite many more than the planned 2960 authors if they want to have 25% of 296 participants in each category (Bachelors, Master’s, doctoral, professorship).*

We are very pleased that we could address your doubts regarding the sampling bias. Thank you for voicing your concern about our planned recruitment strategy. We amended our invitation strategy in the following way: In the second invitation wave, we will consider the actual response rate of the first invitation wave to determine how many more participants of open quotas need to be invited. This is described in the section *data collection and stopping rule*: “If this does not result in a sufficient sample size, an additional invitation wave will be conducted. In this second wave, participants will subsequently be invited for quotas that have not yet been filled. For each open quota, the response rate of the first invitation wave will be used to calculate how many more participants need to be invited to fill the quota.” (p. 13 f, l. 262 ff) and in more detail in the Supporting Information (see S2 Text): “To determine how many people need to be recruited in the second wave to fill the quotas, we will use the response rate from the first invitation wave. This can be illustrated by an example: Since we are aiming for 25% quotas, our final sample should contain *n* = 74 participants from each quota (25% of our targeted sample size *N* = 296). If after the first invitation wave, only 64 members of a quota have participated, this would mean that ten people are still missing in this quota. To calculate how many more participants need to be invited to fill the quota, the overall response rate of the first invitation wave will be used: If only 237 (instead of 296) of the 2960 invited people have participated, this would correspond to an actual response rate of 8% instead of the estimated 10%. If we assume that 8% of invited researchers will also respond in the second invitation wave, we would need to invite 125 more participants to fill our example quota (since 10 is 8% of 125). Calculations of this kind will be done for all open quotas after the first invitation wave, to determine how many more participants to invite. However, due to reasons of practicality, no more than 740 more participants (i.e., 25% of the first invitation wave) will be invited per quota in the second wave.”.

*For all hypotheses 1.1-1.6, it could be interesting to see if the variable “have used preregistration in the past” could account for the effects you might find. We can’t establish causality with these hypotheses (i.e., whether the intention to register causes the beneficial attitudes or vice versa). If you find an effect, but you also find that this effect is eliminated when accounting for having used preregistration in the past, than another potential explanation of the results could simply be “those who registered in the past have more beneficial attitudes towards preregistration AND a greater intention to preregister again”.*

Thank you for your comment. In case the control variable “preregistration experience” is significant, we will conduct additional exploratory analyses to further investigate potential directions of effects. If these result in the conclusion “those who registered in the past have more beneficial attitudes toward preregistration AND a greater intention to preregister again,” we would be able to conclude that preregistering in the past does not discourage one from preregistering in the future (i.e., we can rule out that researchers have had negative experiences that would discourage them from preregistering again), which we think would also be a valuable information.

*Other comments.*

*I’m not familiar with the term “habilitation”.*

Habilitation is a qualification required to obtain a professorship in many European countries. To allow individuals unfamiliar with habilitation to answer the question, "full professorship" is also included in the response: “Habilitation and/or full professorship”.

*[line 275] Will “psychology” be used as a term in the search query box and if so within which fields (e.g., Title, keywords, abstract), or will it be used as a filter that the websites allow you to select from.*

*[L 235: The term “psychology” will be searched for on specified databases, and resulting hits will be sorted from newest to oldest.]*

The former. To provide all details while still keeping the description reasonably concise, we included this information in the Supporting Information (see S2 Text): “On Web of Science, PubMed, PSYNDEX, and PsycInfo, the search term will be set to search “all fields”, it will be searched for the keyword “psychology”, and documents will be set to articles, to get a broad image of research articles focusing on psychology. Documents will be sorted from new to old. All articles that do not focus on psychology will be excluded. On OSF Registries, it will also be searched for the keyword “psychology” and documents will be sorted from new to old.”

*I suggest you keep the phrase you deleted, it is relevant information. “(17 PhD students, three postdocs, seven professors, and two members of other academic groups which were screened out)*

*[L 550 f: In this time, 29 participants started the survey of which 20 completed it, yielding an overall response rate of 10%.]*

We added this part of the sentence again in the manuscript: “In this time, 29 participants (17 PhD students, three postdocs, seven professors, and two members of other academic groups which were screened out) started the survey of which 20 completed it (14 PhD students and 6 professors), yielding an overall response rate of 10%.” (p. 27, l. 553 ff).

*The new question in response to Reviewer 4 still does not solve the issue they raise: [We changed the option to “Preregistration aims at increasing the transparency of potential changes made to a study.” to reduce ambiguity.] Some people may disagree or see this as a less important purpose of preregistration. For example, other reasons are to share what research is being done to reduce duplication or to help develop solid analysis plans.*

We understand that there might be different approaches of how to define preregistrations, and what are important aspects of preregistration. This is precisely why we have chosen to present a definition in our survey, so that all participants know what aspects of preregistration we are referring to and to ensure consistent understanding. We have made it clear in the survey that the knowledge check specifically targets the understanding of our presented definition (“Which aspects belong to the definition of “preregistration”, based on the definition on the previous page? This question’s purpose is to check if you understood our definition of “preregistration” correctly. There are, of course, many ways to define preregistration. However, please base your answer on our definition and keep this definition in mind when answering the survey. Please select every correct option.”). However, even if participants answer this question incorrectly, they are not excluded but are only shown the definition again, and are then redirected to the main survey.

***Reviewer #4***

*The authors have done an impressive job at responding to all author comments and I thank them for responding to all of my comments so completely. I still have reservations about the theoretical basis of the study and the validity of the measures but, thanks to the authors responses, I look forward to the results of the study.*

Thank you for your review, which has helped us immensely to improve our study. We agree that there are some limitations which we will fully disclose when discussing the results of our study.
